# Structure-Activity Relationship of Nerve-Highlighting Fluorophores

**DOI:** 10.1371/journal.pone.0073493

**Published:** 2013-09-09

**Authors:** Summer L. Gibbs, Yang Xie, Haley L. Goodwill, Khaled A. Nasr, Yoshitomo Ashitate, Victoria J. Madigan, Tiberiu M. Siclovan, Maria Zavodszky, Cristina A. Tan Hehir, John V. Frangioni

**Affiliations:** 1 Department of Medicine, Beth Israel Deaconess Medical Center, Boston, Massachusetts, United States of America; 2 Advanced Imaging Research Center, University of Texas Southwestern Medical Center, Dallas, Texas, United States of America; 3 Diagnostics and Biomedical Technologies, GE Global Research, Niskayuna, New York, United States of America; 4 Department of Radiology, Beth Israel Deaconess Medical Center, Boston, Massachusetts, United States of America; University of East Anglia, United Kingdom

## Abstract

Nerve damage is a major morbidity associated with numerous surgical interventions. Yet, nerve visualization continues to challenge even the most experienced surgeons. A nerve-specific fluorescent contrast agent, especially one with near-infrared (NIR) absorption and emission, would be of immediate benefit to patients and surgeons. Currently, there are only three classes of small molecule organic fluorophores that penetrate the blood nerve barrier and bind to nerve tissue when administered systemically. Of these three classes, the distyrylbenzenes (DSBs) are particularly attractive for further study. Although not presently in the NIR range, DSB fluorophores highlight all nerve tissue in mice, rats, and pigs after intravenous administration. The purpose of the current study was to define the pharmacophore responsible for nerve-specific uptake and retention, which would enable future molecules to be optimized for NIR optical properties. Structural analogs of the DSB class of small molecules were synthesized using combinatorial solid phase synthesis and commercially available building blocks, which yielded more than 200 unique DSB fluorophores. The nerve-specific properties of all DSB analogs were quantified using an *ex vivo* nerve-specific fluorescence assay on pig and human sciatic nerve. Results were used to perform quantitative structure-activity relationship (QSAR) modeling and to define the nerve-specific pharmacophore. All DSB analogs with positive *ex vivo* fluorescence were tested for *in vivo* nerve specificity in mice to assess the effect of biodistribution and clearance on nerve fluorescence signal. Two new DSB fluorophores with the highest nerve to muscle ratio were tested in pigs to confirm scalability.

## Introduction

Nerve damage during surgery results in significant morbidity for patients, causing both chronic pain and permanent paralysis [1–3]. Nerve-sparing surgery can prove difficult, as currently no nerve-specific contrast agents are clinically available to aid in intraoperative visualization. At present, nerve detection during surgery is largely completed through electromyographic (EMG) monitoring in delicate areas, such as surgical procedures near the larynx, thyroid, or spinal cord [4–6], or direct visualization by the surgeon. Current methods are suboptimal as EMG monitoring is an electrical stimulus detection method rather than an imaging methodology, and direct visualization can be hampered by the nature of the small, translucent nerve structures that are typically protected deep within the tissue. Improved nerve visualization would result from a nerve-specific optical contrast agent that could aid nerve visualization during image-guided surgery.

Fluorescence-guided surgery is quickly gaining traction because it provides real-time assessment of normal and diseased tissues. A number of fluorescent image-guided surgery systems are in development, clinical trials, or are commercially available for use [7]. However, targeted fluorophore availability is currently limited, and a clinically viable nerve-specific fluorophore does not exist. Histopathological examination of myelin has been possible for many years using a number of colorimetric stains [8–10], and fluorophores that specifically label myelin have also been developed [11,12]. However, none of the currently available histopathological contrast for myelin can be administered systemically to stain nerve tissue *in vivo*, because the contrast agents will not penetrate the blood nerve barrier (BNB).

There are only four classes of fluorescent molecules that have been found to penetrate the BNB and stain nerve tissue *in vivo* following systemic administration, which include nerve-specific peptides and three classes of small molecule organic fluorophores. The nerve-specific peptides are a targeting sequence that largely highlights the epineurium with some binding to the endoneurium [13]. The three classes of nerve-specific small molecule organic fluorophores include the stilbene derivatives [14], the distyrylbenzene (DSB) derivatives [14–17], and the styryl pyridinium (FM) fluorophores [18,19]. The FM dyes have been found to stain only the dorsal root and trigeminal ganglia when administered systemically [18]. The stilbene derivatives highlight all nerve tissues when administered systemically, but currently have ultraviolet (UV) excitation with UV to blue wavelength emission [14]. In the current study, the DSB class of fluorophores was chosen for further study. Although DSBs highlight all nerve tissues when administered systemically, they absorb and emit in the UV and visible, respectively, and have suboptimal biodistribution properties [16]. The hypothesis underlying this study was that the combination of synthesis of DSB analogs, quantitative QSAR modeling, and large animal validation would reveal the pharmacophore that mediates nerve-specific binding, thus enabling optimization of molecules for future clinical translation.

## Results

### Distyrylbenzene Library Synthesis

**Figure 1:**
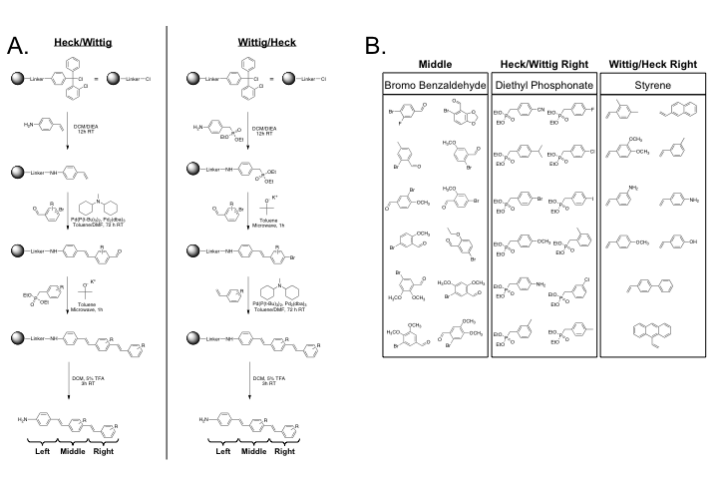
Combinatorial solid phase synthetic scheme and building blocks of DSB library. (**A**) Chlorotrityl chloride (CTC) resin was used to synthesize the DSB library using optimized Heck and Wittig reactions. The library was synthesized using either the Heck reaction followed by the Wittig reaction (Heck/Wittig = 120DSB fluorophores) or the Wittig reaction followed by the Heck reaction (Wittig/Heck = 110DSB fluorophores). The left portion of the molecule was loaded onto the CTC resin followed by carbon-carbon bond formation between the building blocks using the optimized Heck and Wittig reactions. The molecules were cleaved from the CTC beads following reaction completion using a mixture of 5/95 TFA/DCM. (**B**) The commercially available building blocks utilized to synthesize analogs of DSB lead structure at the middle and right portions of the molecule are shown.

The DSB fluorophore library was synthesized using the previously characterized nerve-specific structure 4,4’-[(2-methoxy-1,4-phenylene) di-(1E)-2,1-ethenediyl] bis-benzenamine (BMB) as the lead compound from which analogs were derived [16]. Solid phase combinatorial synthesis was utilized to create 230 structural analogs of BMB (Table S1) using commercially available building blocks (Table S2). To facilitate a modular assembly of the library, the DSB structure was divided into left, middle, and right moieties (Figure 1A), assembled from commercially available building blocks (Figure 1B) via efficient carbon-carbon bond forming reactions. The carbon-carbon bonds between the building blocks were formed utilizing an optimized Horner-Emmons-Wittig reaction (subsequently referred to as the Wittig) and Heck reaction [20–23]. The library was synthesized using either the Wittig reaction followed by the Heck reaction or the Heck reaction followed by the Wittig reaction (Figure 1A). Two left molecules were used for synthesis and loaded onto commercially available chlorotrityl chloride (CTC) resin, including diethyl 4-aminobenzylphosphonate for the Wittig/Heck reaction and 4-aminostryrene for the Heck/Wittig reaction (Figure 1A). The same 12 middle molecules were used for both the Heck/Wittig and Wittig/Heck synthetic schemes, as all middle molecules contained both reactive groups (Figure 1B). The diethyl phosphonate building blocks were used as the right moiety for the Heck/Wittig reaction while the styrene building blocks were used as the right moeity for the Wittig/Heck reaction (Figure 1B). All DSB analogs were cleaved following reaction completion from the CTC resin and identified by molecular weight using liquid chromatography/mass spectroscopy (LC/MS) analysis for reaction completion, purity analysis, and yield (Table S3). The partition coefficient (LogD at pH = 7.4) for each of the 230 DSB fluorophores was calculated (Table S3).

### Characterization of Spectroscopic Properties

The absorbance spectrum of each new compound was collected during LC/MS analytical evaluation using the inline photodiode array detector (PDA) with full spectral capabilities. The absorbance spectral information was extracted from the product peak (Table S4). Purity analysis was completed using the photodiode array (PDA) spectrum where the area under each peak was calculated to find the total area for all compounds in the crude mixture. The area under the product peak was used to determine the purity of the sample and yield in milligrams. Purity information was used to adjust the amount of dimethyl sulfoxide (DMSO) necessary to dissolve the product in the crude mixture at 100 mM to ensure equivalent product concentration for each stock solution. Each crude fluorophore was diluted from the stock solution to 10µM concentration in DMSO. The emission spectrum of each new fluorophore was recorded following excitation at 350, 375, 400, 425, and 450 nm (Table S4). Compounds found to be positive for nerve-specific fluorescence by *ex vivo* screening (explained as follows) were purified by preparative-HPLC; after which additional spectral data were collected in DMSO, methanol (MeOH), and fetal bovine serum (FBS) at 10 µM. Absorbance spectra were collected and used to determine the maximum excitation wavelength used for emission spectra collection (Table S4).

### Ex Vivo Nerve-Specific Fluorescence Library Screening

**Figure 2:**
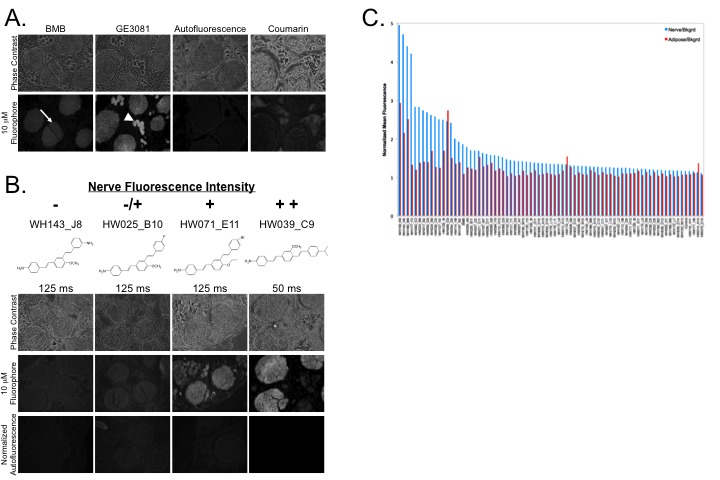
*Ex Vivo* Nerve-Specific Fluorescence Assay. Nerve-specific fluorescence intensity was determined through staining of pig sciatic or brachial plexus nerves cut in cross section. (**A**) The previously characterized nerve-specific DSB contrast agents BMB and GE3081 were used as positive control. Nerve tissue was incubated with IV formulation not containing fluorophore (negative control, autofluorescence only) and with a fluorophore not specific for nerve tissue (coumarin). The *ex vivo* nerve-specific fluorescence assay showed nerve (arrow) and adipose (arrow head) accumulation of the incubated fluorophores. (**B**) The spectrum of nerve-specific fluorescence intensity is shown for 4 representative compounds where name and chemical structure are also shown. Phase contrast images of the nerve tissue, fluorescence images following incubation with 10 µM DSB fluorophore, and control autofluorescence images with equivalent exposure time and normalization are shown. The quantitative scale used is as follows: = nerve fluorescence equivalent to control autofluorescence, -/+ = nerve fluorescence lower than BMB, but higher than control autofluorescence, + = nerve fluorescence equivalent to BMB with the same exposure time, + + = nerve fluorescence brighter than BMB with lower exposure time required for imaging. (**C**) Nerve to background (N/B) and adipose to background (A/B) ratios were quantified for all 75 purified DSB fluorophores using region of interest analysis on three regions per image. Of note, 16 compounds showed higher N/B fluorescence than BMB, while only 3 compounds had fluorescence below the control autofluorescence. All but 3 compounds had greater N/B fluorescence than A/B fluorescence.

All fluorophores were screened for nerve-specific fluorescence using pig brachial plexus or sciatic nerve tissue from unrelated experiments cut in cross section. Nerve tissue sections were incubated with each crude fluorophore [24] at 1 mM, 100 µM, and 10 µM, where purity information obtained during initial characterization was used to adjust the amount of DMSO necessary to dissolve the product in the crude mixture at the same stock concentration ensuring that the same amount of novel fluorophore was incubated with each nerve section for *ex vivo* screening. Following incubation with the previously known nerve-specific fluorophores, homogenous fluorescence was seen throughout the nerve bundle, which was significantly higher than the background autofluorescence (Figure 2A). Additionally, adipose partitioning was also seen for compounds with increased LogD (Table S3) where GE3081 (LogD=5.5) showed higher adipose fluorescence than BMB (LogD=4.8). The *ex vivo* nerve-specific screen provided nerve-specific and adipose-specific fluorescence information for all compounds. Nerve sections incubated with fluorophores not specific for nerve tissue did not demonstrate nerve-specific fluorescence signal (Figure 2A).

Nerve fluorescence signal from all new DSB fluorophores was compared to nerve-specific fluorescence seen from BMB incubated nerve tissue sections and autofluorescence from vehicle incubated nerve tissue sections. *Ex vivo* nerve-specificity screening of the 230 compound DSB library demonstrated a spectrum of nerve-specific fluorescence (Figure 2B), which was qualified on a 4-point scale (-, -/+, +, or + +, Table S5) as compared to the nerve-specific fluorescence of BMB (Figure 2A). Representative nerve-specific fluorescence data for the crude compound screen is shown in Figure 2B where all images were acquired with equal exposure time and displayed with equal normalization. All crude compounds were screened at each concentration in triplicate.

To ensure validity of all hit compounds from the crude compound screen, all compounds that showed nerve fluorescence signal similar to BMB or higher in the crude compound screen (+ or + +) were purified using preparative-HPLC and screened again using the same *ex vivo* nerve-specificity assay [25–27]. For all purified compounds, the *ex vivo* nerve-specific assay was completed at 10 µM concentration in triplicate and qualified (Table S5). Additionally, the nerve to background (N/B) and the adipose to background (A/B) ratios were quantified using region of interest analysis on three representative regions in each image (Figure 2C). In total, 75 compounds were purified and re-screened for nerve-specific fluorescence, where 16 compounds showed nerve-specific fluorescence higher than BMB, and all but 3 compounds showed higher nerve-specific fluorescence than control autofluorescence (Figure 2C). In general compounds had higher N/B than A/B ratio, with only 3 compounds showing higher A/B than N/B ratio.

### Quantitative Structure-Activity Relationship Modeling

**Figure 3:**
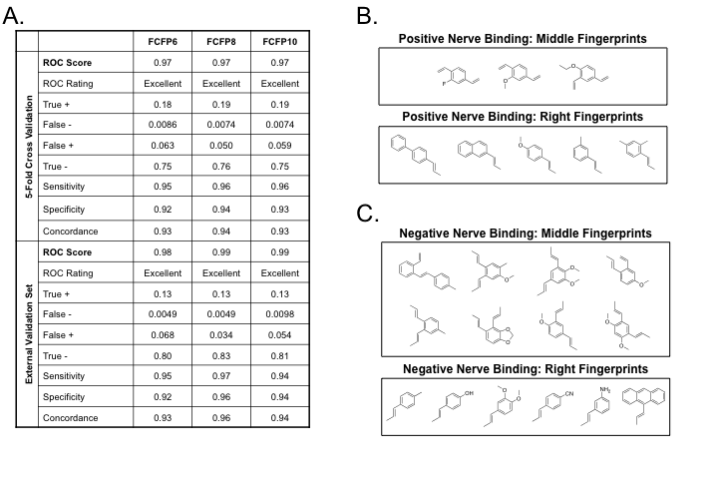
Quantitative Structure-Activity Relationship (QSAR) Modeling and DSB Pharmacophore Definition. (**A**) The performance of the QSAR model using the purified *ex vivo* nerve-specific fluorescence assay are demonstrated with three differing fingerprint types. The model descriptors are averages over 5 runs of 5-fold cross-validation on the training dataset and testing on the external test set. The Receiver Operator Characteristic (ROC) curve analysis was used to assess model performance. The ROC score represents the area under the ROC curve, which defines the fraction of true positive and false positive compounds. ROC scores range from 0.5 to 1.0, where 1.0 shows perfect model selectivity and a score of 0.5 corresponds to random classification. The QSAR models are rated as “fair” if the ROC score is between 0.7 to 0.8, “good” if the ROC score is between 0.8 to 0.9, and “excellent” if the ROC score is above 0.9. Additional properties characterizing the model are sensitivity, specificity, and concordance defined as a function of the number of true positives (TP), true negatives (TN), false positives (FP), and false negatives (FN) as follows: sensitivity = TP/(TP + FN), specificity = TN/(TN + FP), and concordance = (TP + TN)/(TP + TN + FP + FN). The most relevant fingerprints from the QSAR model for both the middle and right rings predicted to (**B**) positively and (**C**) negatively influence nerve-specific fluorescence intensity.

The *ex vivo* nerve-specific screening data were used to generate a quantitative structure-activity relationship (QSAR) model for nerve-specific fluorescence. Only those DSB fluorophores showing *ex vivo* nerve-specific fluorescence after purification were considered positive/active in the QSAR model. Model quality was evaluated based on the model’s ability to distinguish between compounds with and without nerve-specific fluorescence (active and inactive, respectively) during both a 5-fold cross-validation and validation performed on an external test data set. Performance properties of the models generated for the DSB fluorophore library with 3 different fingerprint lengths (FCFP6, FCFP8, and FCFP10) are summarized in Figure 3A. The average Receiver Operator Characteristic (ROC) scores were all above 0.9 and rated as excellent for the 5-fold cross-validation on the training set, as well as for the validation on the external test data set. Increasing the fingerprint length resulted in only slight improvements in ROC score (Figure 3A). The chemical fingerprints that contribute both positively and negatively to nerve-specific fluorescence signal were determined for both the middle and right building blocks (Figure 3B and 3C), where chemical moieties were varied on the benzene ring as compared to the lead DSB structure.

### In Vivo Biodistribution of Nerve-Specific Fluorophores

**Figure 4:**
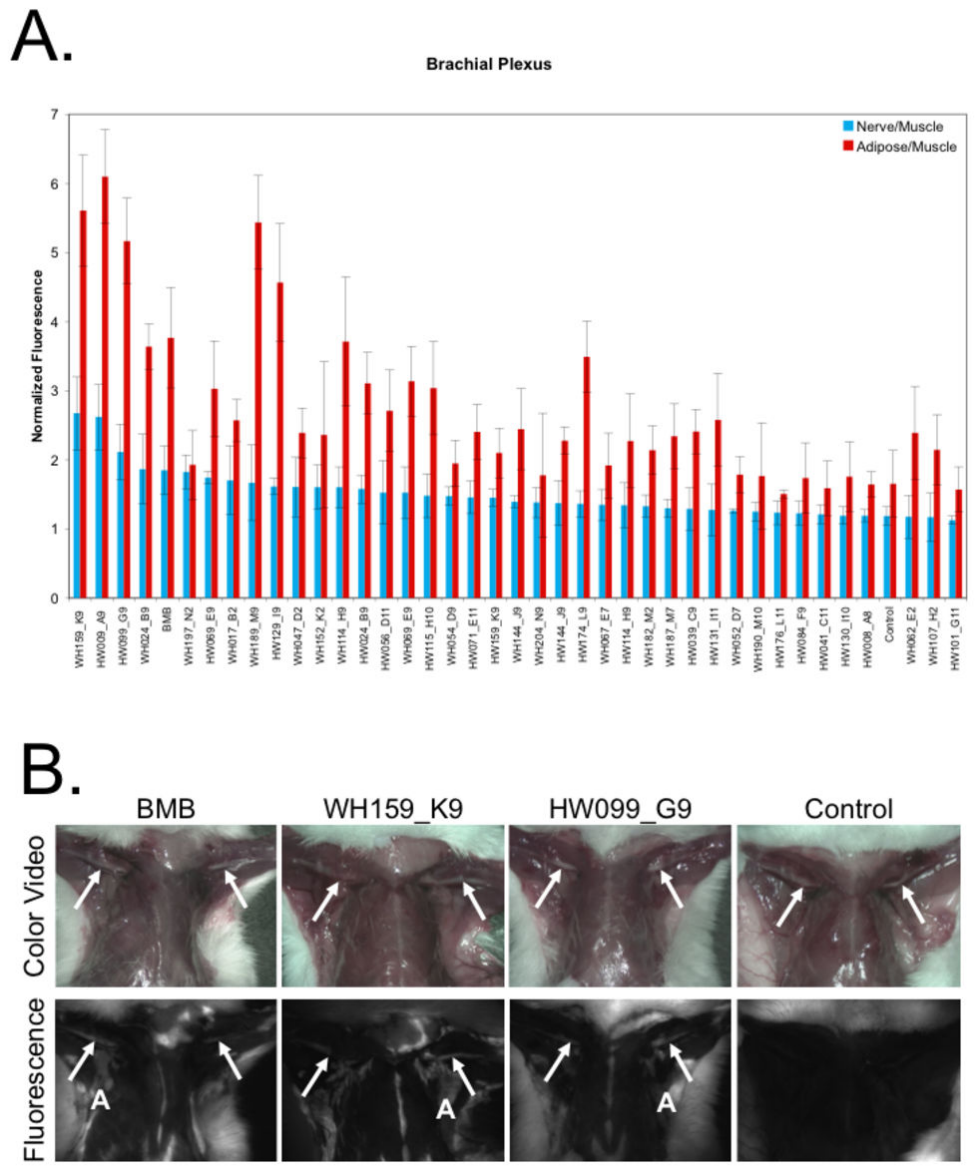
*In Vivo* Biodistribution and Nerve-Specific Partitioning. All purified compounds positive by *ex vivo* nerve-specific screening were screened *in vivo* in mice. The (**A**) brachial plexus nerve was quantified using region of interest analysis for n=3 mice per compound. The average nerve to muscle (N/M) and adipose to muscle (A/M) fluorescence ratios normalized to the exposure time of the image were calculated. The error bars represent standard deviation calculated over the 3 animals for n=6 brachial plexus nerves. (**B**) Representative images of the brachial plexus for the 2 new compounds with highest N/M ratio (WH159_K9 and HW099_G9) are shown compared to BMB and vehicle injected control autofluorescence. The arrows point to the brachial plexus in all images, except the control autofluorescence image where the brachial plexus is not visible. Representative adipose tissue is denoted in each fluorophore injected mouse with an ‘A’.

All purified fluorophores that showed positive nerve fluorescence by the *ex vivo* nerve-specific assay were administered to mice for *in vivo* biodistribution studies to determine nerve partitioning and retention properties. Additionally, to ensure the validity of excluding compounds negative for nerve fluorescence by the *ex vivo* nerve-specificity assay, the compounds with chemical structure most similar to BMB (WH047_D2 – WH060_D15) and GE3082 (HW006_A6 – HW015_A15) were also purified and screened *in vivo* (Tables S1 and S5). All purified compounds were initially screened in n=1 mouse where imaging was performed 4 hours after intravenous (IV) administration of the fluorophore, based on previously demonstrated pharmacokinetics of DSB fluorophores [16]. Images of the brachial plexus, sciatic, trigeminal ganglia, and optic nerves were quantified for each mouse and compared to the mean autofluorescence signal from 5 vehicle injected control mice. Any fluorophore with nerve to muscle (N/M) ratio greater than 1 standard deviation above the mean vehicle injected control N/M ratio was administered to two additional mice totaling n=3 tested animals for all compounds positive for *in vivo* nerve fluorescence. The mean and standard deviation of the N/M and adipose to muscle (A/M) ratios were calculated for each nerve site across all animals. The mean and standard deviation of nerve fluorescence of n=3 mice is shown in the brachial plexus (Figure 4A), sciatic, trigeminal ganglia, and optic nerves (Figure S1). The two new DSB fluorophores with highest mean N/M ratio in the brachial plexus are demonstrated in comparison to a BMB injected animal and a vehicle injected control animal (Figure 4B). Even though it showed one of the highest N/M ratios, compound HW009_A9 was not chosen for further study, as it is the previously studied GE3082 synthesized as part of the DSB library. All compounds with a mean N/M ratio within 1 standard deviation of the control N/M ratio were quantified in a single mouse (Figure S2).

### Ex Vivo Human Nerve Cross Reactivity

**Figure 5:**
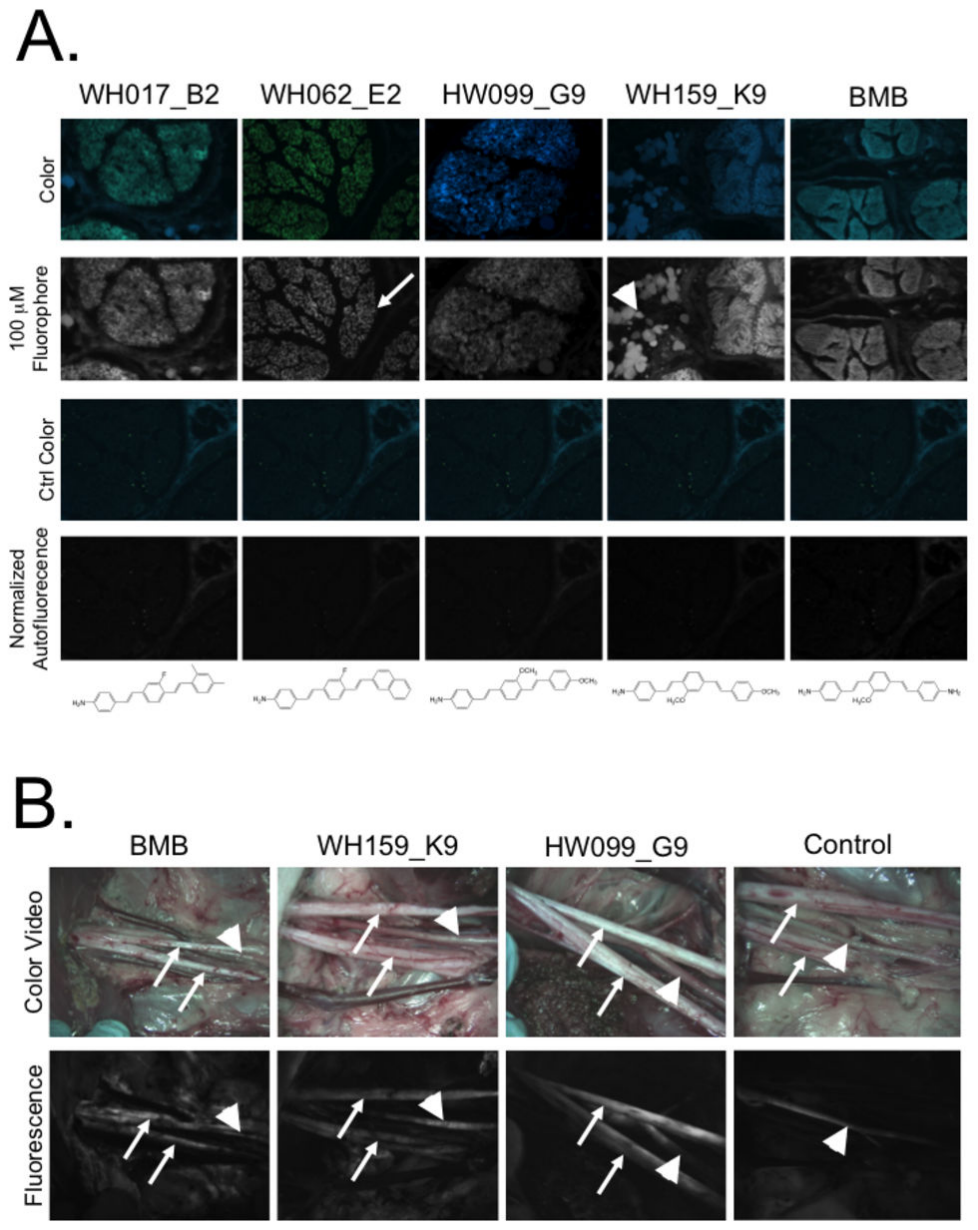
DSB library cross reactivity and scalability. (**A**) All purified compounds positive by *ex vivo* nerve-specific fluorescence screening were screened for cross reactivity in human sciatic nerve tissue. Five representative examples of nerve-specific fluorescence are shown where fluorescence of the compound incubated with the nerve tissue at 100 µM can be compared to the normalized control autofluorescence. Spectral information for both the fluorophore incubated tissue and the control tissue autofluorescence were acquired using a color camera at the same exposure time and normalization. A representative nerve bundle in cross section is denoted by an arrow in the image labeled WH062_E2. Representative lipid droplets are denoted by an arrowhead in the image labeled WH159_K9. (**B**) Swine brachial plexus nerve images of animals administered BMB, WH159_K9, HW099_G9, and IV formulation 4 hour prior to imaging are shown. Color video of the surgical field of view and nerve-specific fluorescence using a 550/50 nm BP filter are shown for each compound and control autofluorescence. A branch of the brachial plexus nerve is denoted by the arrow. The autofluorescent artery is shown with an arrowhead.

To ensure DSB fluorophores exhibit cross reactivity among species, *ex vivo* nerve staining was completed on human sciatic nerve tissue. All purified fluorophores with positive *ex vivo* pig nerve fluorescence were screened in human sciatic nerve at 10 µM and 100 µM. All compounds with positive binding in pig nerve also demonstrated positive binding in human sciatic nerve. Representative human nerve tissue sections are shown imaged with both a conventional fluorescence camera as well as a color camera to highlight the visible spectral differences among fluorophores. Color and fluorescence images of the autofluorescence from the nerve tissue incubated with vehicle only, imaged with the same exposure time and normalized equally to the fluorescence images, are also shown (Figure 5A). Some compounds exhibited hypsochromic (blue-shifted) fluorescence in adipose tissue as compared to nerve tissue (WH017_B2), while others showed little adipose fluorescence accumulation (WH062_E2) and some demonstrated similar emission wavelengths in both adipose and nerve tissues (WH159_K9).

### Swine Nerve Imaging

Two new fluorophores from the DSB library were chosen for additional study in swine. WH159_K9 was selected for swine nerve imaging because it showed the highest N/M ratio in mouse biodistribution studies in the brachial plexus and sciatic nerve (Figure 4A and Figure S1). As a comparison, a close structural analog specifically HW099_G9, was also selected for swine imaging. Each new fluorophore was administered IV to a Yorkshire pig and imaged 4 hours after administration in the brachial plexus nerve. As a positive control for the imaging study, BMB was also administered to a pig and imaged in the brachial plexus nerve 4 hours after IV administration. To determine the contribution of tissue autofluorescence in the wavelength range used for imaging, the brachial plexus nerve of a vehicle injected control animal was imaged 4 hours after vehicle administration. Both new compounds show nerve-specific fluorescence similar to that of BMB. Strong autofluorescence signal (presumably from elastin) was seen in the major arteries of all four animals (including the control animal), whereas no nerve-specific fluorescence was seen in the control animal (Figure 5B).

## Discussion

Nerve damage resulting from surgery causes significant morbidity for patients, which could be mitigated through improved visualization. Although fluorescent image-guided surgery systems exist, a nerve-specific fluorophore has proven difficult to develop. Currently, only three classes of small molecule structures with the ability to penetrate the BNB and stain nerve tissue *in vivo* are known and none are optimal for clinical translation. Development of a NIR nerve-specific small molecule fluorophore would be ideal, but is hindered by the fact that the structure-activity relationship (SAR) for nerve-specific fluorescence has not been previously quantified. In the work presented herein, 230 analogs of a promising nerve-specific fluorophore lead structure previously shown to highlight all nerve tissue in mice, rats, and pigs following systemic administration [16] were synthesized and screened for nerve-specific fluorescence. Results were used to build a QSAR model to define the pharmacophore for nerve-specific fluorescence and direct future synthetic efforts to develop an optimized NIR nerve-specific fluorophore for clinical translation.

The results of the QSAR modeling revealed that the overall configuration of the three-ring system was the dominant feature that influenced nerve-specificity. The chemical fingerprint features characterizing the middle ring (Figure 3B and 3C) had the highest contributions to nerve-specific fluorescence, both positively and negatively. The most favorable compounds for nerve-specific fluorescence were those with a para-configuration around the middle ring. Of the 204 unique compounds tested, 30 had a para-configuration and 27 demonstrated nerve-specific fluorescence. The ortho-configuration was unfavorable resulting in compounds lacking nerve specificity, where all 70 ortho-configuration compounds were negative for nerve-specific fluorescence. The nerve-specificity of compounds with a meta-configuration depended on the existence and nature of the additional substituents, mainly on the middle ring, where the presence of two methoxy groups on the middle ring resulted in inactive compounds (Figure 3C). Of the 104 compounds with meta-configuration of the middle ring, only 10 were found to be active. Therefore the QSAR model results demonstrate the pharmacophore for the lead DSB structure requires an extended para-configuration with limited substituents on the middle benzene ring to ensure nerve-specific fluorescence.

Adipose tissue accumulation of the current DSB fluorophores is an undesirable property of the lead structure, which varies significantly from compound to compound. Of note, when adipose tissue accumulation was quantified *in vivo*, all compounds showed higher A/M fluorescence than N/M fluorescence (Figure 4A). By contrast, *ex vivo* quantification of N/B and A/B fluorescence showed higher N/B ratio for 72 out of the 75 screened compounds (Figure 2C). This difference in fluorescence signal can be attributed to two possible phenomena. First, there may be a difference in compound partitioning when administered systemically *in vivo* versus incubation on a 10 µm thick tissue section, which can be attributed to *in vivo* biodistribution and/or clearance of the compound rather than to an inherently higher adipose tissue-specificity as compared to nerve-specificity. Second, the thickness of the imaged tissue is not equivalent in the two situations; the adipose tissue thickness *in vivo* is far greater than the nerve tissue thickness. Thus, the fluorescence signal from the adipose tissue is integrated over many thicknesses as compared to that of the nerve fluorescence signal, causing the A/M signal to appear inflated as compared to that of the N/M signal when quantified from the *in vivo* images. Intriguingly, some DSB fluorophores demonstrate adipose fluorescence at a different peak wavelength than nerve fluorescence providing the opportunity to spectrally separate fluorescence signals if adipose tissue accumulation cannot be entirely eliminated from the DSB pharmacophore (Figure 5A). The importance of off-target adipose accumulation is currently under investigation through additional QSAR modeling to define this specific SAR.

Although none of the current DSB fluorophores are ideal for nerve-specific image-guided surgery, cross-species reactivity was demonstrated and a pharmacophore for nerve-specific fluorescence was defined. The SAR is largely based on the configuration of the pharmacophore rather than presence or absence of specific substituents on the benzene rings. The SAR also demonstrates a limitation to the current study in that the DSB library is relatively small, with just over 200 unique compounds, and thus may not contain enough variability to unequivocally determine the substituents that positively and negatively influence nerve-specific fluorescence. In future studies, this known SAR will be utilized to direct synthesis of additional libraries of fluorophores to both further refine the QSAR model and work towards synthesis and characterization of a clinically viable red-shifted nerve-specific fluorophore for image-guided surgery. Chemical strategies currently under investigation to red-shift the excitation and emission of the DSB pharmacophore include adding structural rigidity around the central benzene ring, maximizing the push-pull nature of the chemical moieties on the two terminal benzene rings using zwitterionic species, and employing the structural motifs consistent with near infrared cyanine dyes into the pharmacophore.

## Materials and Methods

### DSB Library Synthesis, Characterization, and Purification

The DSB fluorophore library containing 230 DSB fluorophores (Table S1) was synthesized on 2-chlorotrityl chloride (CTC) resin (1.5 mmol/gram, 100-200 mesh, Novabiochem 1% DVB, Figure 1A) using commercially available building blocks (Sigma Aldrich, Acros Organics, Maybridge Chemicals, and TCI America, Figure 1B and Table S2). All solvents and catalysts were purchased from Sigma Aldrich and used without further purification. All building blocks with exception of those with terminal amino groups were used as purchased. Diethyl (4-aminobenzyl) phosphonate, 3-aminostyrene, and 4-aminostyrene were protected with a tert-butyloxycarbonyl (BOC) group prior to their use as a right-side building blocks. The BOC protection reaction was performed using conventional synthetic procedures and is explained briefly as follows. The terminal amino containing building block (20.5 mmol) was dissolved in a mixture of tetrahydrofuran (THF)/water (65 ml THF/16 ml water, 80/20 [v/v]). Di-tert-butyl dicarbonate (4.90 g, 22.5 mmol) and sodium bicarbonate (2.58 g, 30.75 mmol) were added and the reaction mixture was stirred at room temperature for 20 h. to the reaction mixture. The organic solvent was evaporated *in vacuo* and the aqueous layer was extracted with ethyl acetate (3 x 50 ml). The combined organic phase was washed with 10% citric acid and saturated sodium carbonate brine (3 x 50 ml), dried over anhydrous sodium sulfate, and filtered. The solvent was evaporated *in vacuo* and the crude product was recrystallized from hot hexanes to give the desired product as white crystals.

Formation of carbon-carbon bonds between building blocks was completed using an optimized Horner-Emmons Wittig reaction (subsequently referred to as the Wittig reaction) and Heck reaction optimized for solid phase synthesis [20–23]. The library was synthesized using the Heck reaction followed by the Wittig reaction or using the Wittig reaction followed by the Heck reaction yielding 230 DSB analogs total (Figure 1A). For each Heck/Wittig reaction sequence, 50 mg of CTC resin was loaded with 4-aminostyrene. 10 molar equivalents (calculated from the CTC loading of the resin) of diisopropalethylamine (DIEA, 0.124 ml, 0.75 mmol) were added to 10 molar equivalents of 4-aminostryene (0.0894 g, 0.75 mmol) dissolved in dichloromethane (2 ml). The mixture was vortexed for 10 minutes and then poured over the CTC beads followed by vortexing at room temperature overnight. The beads were subsequently washed using polypropylene (PP) 10 ml filtered syringes (Torviq, Niles, MI) with a series of solvents as follows: dichloromethane (DCM), dimethylformamide (DMF), methanol (MeOH), DMF, DCM, ethyl ether (3 washes/solvent, 10 ml/wash). Due to the oxygen and moister sensitivity of the optimized Heck and Wittig reactions all reactions were carried out under an inert atmosphere in a glove box (Unilab 1200, MBraun Inc., Stratham, NH). Initial reaction conditions, including molar equivalents of reactants and catalysts, for the Heck reaction were optimized for solid phase synthesis from a previously published procedure [22]. The loaded CTC beads were suspended in DMF (0.5 ml) to which 10 molar equivalents of *N*,*N-*dicyclohexylmethylamine (0.784 mmol, 0.168 ml) was added. 10 molar equivalents of the desired bromo benzaldehyde building block (0.698 mmol, Figure 1B) were dissolved in anhydrous toluene (0.5 ml) to which 10 molar equivalents of bis(tri-t-butylphosphine) palladium(0) (0.011 mmol, 5.4 mg) and 10 molar equivalents of tris(dibenzylideneacetone) dipalladium(0) (0.0052 mmol, 4.8 mg) were added. The bromo benzaldehyde mixture was added to the CTC resin mixture and vortexed at room temperature for 72 hours after which they were washed using PP 10 ml filter syringes with the following solvents: water, DMF, DCM, DMF, DCM, ethyl ether (3 washes/solvent, 10 ml/wash). The right portion of the molecule was added using an optimized Wittig reaction. 10 molar equivalents of potassium tert-butoxide (1M in THF, 0.75 mmol, 0.093 ml) were added to 10 molar equivalents of the desired diethyl phosphonate building block (0.75 mmol, Figure 1B) dissolved in anhydrous toluene (2 ml). The solution was reacted on a CEM Discover LabMate (CEM, Matthews, NC) microwave reactor under the following conditions: 110°C, 300 Watts, for 1 hour. Following completion of the reaction the beads were washed in a PP 10 ml filtered syringe using the following solvents: water, DMF, DCM, DMF, DCM, ethyl ether (3 washes/solvent, 10 ml/wash). All molecules were cleaved from the CTC beads using a mixture of 5% Trifluoroacetic acid (TFA) and 95% DCM (1 ml/50 mg beads), vortexed at room temperature for 3 hours. The cleaved compounds were filter from the beads, dried using a rotary evaporator, and vacuum dried overnight.

For each Wittig/Heck reaction, 50 mg of CTC resin was loaded with diethyl 4-aminobenzylphosphonate. 10 molar equivalents of DIEA (0.75 mmol, 0.124 ml) were added to 10 molar equivalents of diethyl 4-aminobenzylphosphonate (0.75 mmol, 0.182 g) dissolved in DCM (2 ml). The mixture was vortexed for 10 minutes and then poured over the CTC beads followed by vortexing at room temperature overnight. The beads were washed with the following solvents: DMC, DMF, MeOH, DMF, DCM, ethyl ether (3 washes/solvent, 10 ml/wash). The middle building block was added using the optimized Wittig reaction. 10 molar equivalents of the desired bromo benzaldehyde building block (0.75 mmol, Figure 1B) was dissolved in anhydrous toluene (2 ml), to which 10 molar equivalents of potassium tert-butoxide (1M in THF, 0.75 mmol, 0.093 ml) was added. The bromo benzaldehyde mixture was added to the diethyl 4-aminobenzylphosphonate loaded CTC beads and microwaved using the same conditions. The beads were washed in a PP 10 ml filtered syringe using the following solvents: water, DMF, DCM, DMF, ethyl ether (3 washes/solvent, 10 ml/wash). The right portion of the molecule was added using the optimized Heck reaction. 10 molar equivalents of the desired styrene molecule (0.806 mmol, Figure 1B) was dissolved in anhydrous DMF and 10 molar equivalents of *N*,*N-*dicyclohexylmethylamine (0.840 mmol, 0.180 ml) was added. 10 molar equivalents of each palladium catalyst, bis(tri-t-butylphosphine) palladium(0) (0.011 mmol, 5.8 mg) and tris(dibenzylideneacetone) dipalladium(0) (0.0057 mmol, 5.2 mg) were dissolved in anhydrous toluene (0.5 ml) and added to the diethyl 4-aminobenzylphosphonate loaded CTC beads. The bromo benzaldehyde containing mixture was added to the CTC beads and vortexed at room temperature for 72 hours. The beads were washed using PP 10 ml filter syringes with the following solvents: water, DMF, DCM, DMF, DCM, ethyl ether (3 washes/solvent, 10 ml/ wash). All molecules were cleaved from the CTC beads using a mixture of 5% TFA and 95% DCM (1 ml/50 mg beads), vortexed at room temperature for 3 hours. The cleaved compounds were filter from the beads, dried using a rotary evaporator, and vacuum dried overnight.

All compounds were analyzed for reaction completion and purity using a tandem liquid chromatography/mass spectroscopy (LC/MS) system consisting of a 1525 binary HPLC pump with a manual 7725i Rheodyne Injector (Waters, Milford, MA), a 996 Photodiode Array (PDA) Detector (Waters), and a 2475 Multi-Wavelength Fluorescence Detector (Waters). The column eluate was divided in two using a flow splitter (Upchurch Scientific, Oak Harbor, WA). 80% of the eluate flowed into an evaporative light scatter detector (ELSD, Richards Scientific, Novato, CA), while the rest flowed into a Micromass LCT TOF-ESI spectrometer (Waters) equipped with a Symmetry C18 (4.6 x 150 mm, 5µm particle size) reverse-phase HPLC column. For mass spectrometry, the mobile phase was solvent A=0.1% formic acid in water, solvent B=0.1% formic acid in acetonitrile with a linear gradient from 10% to 100% (from A to B for 30 minutes, flow rate = 1 ml/min, capillary voltage = -3317V, and sample cone voltage = -50V). All compounds were identified by molecular weight, the retention time for each new compound is listed in Table S3. Purity analysis was completed using the PDA spectrum where the area under each peak was calculated to find the total area for all compounds in the crude mixture. The area under the product peak was used to determine the purity of the sample and yield in milligrams. Purity information was used to adjust the amount of dimethyl sulfoxide (DMSO) necessary to dissolve the product in the crude mixture at 100mM.

Following *ex vivo* nerve-specificity screening all compounds positive for nerve-specific fluorescence were purified using reverse phase HPLC purification (HPLC-prep) on a Waters prep-HPLC 150 ml fluid handling unit equipped with a Symmetry Prep C18 column (19 x 150 mm, 7µm particle size), a manual injector (Rheodyne 3725i), and a 2487 dual wavelength absorbance detector (Waters) outfitted with a semi-preparative flow cell. A flow splitter diverted a portion of the eluate into an ELSD with the nebulizer modified to reduce band broadening at low flow rates, while the other portion flowed into a fraction collector (Waters, Fraction Collector II). The ELSD was set to 40°C, with the nitrogen pressure at 3.5 bar and gain of 7. The mobile phase was solvent A=0.1% formic acid in water and solvent B=0.1% formic acid in acetonitrile, a linear gradient from 10% to 100% (from A to B for 45 minutes, flow rate =15 ml/min). All peaks were collected and the product was identified by LC/MS as previously described. Solvent was evaporated *in vacuo*, and vacuum dried overnight.

### DSB Library Spectral Characterization

Absorbance maximum for each crude compound was determined from LC/MS data collection using the spectral capabilities of the PDA (Table S4). A Cary Eclipse fluorescence spectrometer (Agilent, Mattapoisett, MA) with a 96-well plate reader was used to record fluorescence emission spectra. 100 mM stock solutions of crude fluorophores in DMSO were diluted to 10 µM in DMSO. Fluorescence emission spectra were recorded starting 15 nm red shifted from the excitation wavelength to 700 nm using excitation wavelengths of 350, 375, 400, 425, and 450 nm for all compounds (Table S4). All fluorophores with positive *ex vivo* nerve binding were purified by prep-HPLC. Additional spectral measurements were acquired of each purified compound at 10µM in methanol (MeOH), DMSO, and fetal bovine serum (FBS). A Cary 50 Bio UV-Visible spectrophotometer (Agilent) and quartz cuvette were used to collect absorbance spectra in each solvent followed by collection of fluorescence emission spectra (Table S4).

### Ex Vivo Nerve-Specific Fluorescence Library Screening

Sciatic or brachial plexus nerves from swine used in unrelated experiments were harvested, fixed in 2% paraformaldehyde (PFA), and flash frozen in optimal cutting temperature (OCT) compound with liquid nitrogen. Nerves were cryo-sectioned in cross section at 10 µm onto positively charge glass slides. Tissue sections were washed once with phosphate buffered saline (PBS, 2 minutes), fixed with 2% PFA (15 minutes) and washed with PBS (3 x 5 minutes). The previously developed formulation for intravenous (IV) administration was used in the current study to incubate the DSB fluorophores with the nerve tissue [16]. All crude fluorophores were mixed from the 100 mM stock solution into the IV formulation at 1mM, 100 µM, and 10 µM and incubated with the tissue (20 minutes). IV formulation not containing fluorophore was mixed and used to wash each nerve section (2 x 5 minutes) followed by washes with PBS (2 x 5 minutes), after which coverslips were mounted using Fluoromount-G (Southern Biotech, Birmingham, AL).

All slides were imaged on a Nikon TE-300 fluorescence microscope equipped with a mercury excitation source (Chiu Technical Corporation, Kings Park, NY), Orca-ER 12-bit camera (Hamamatsu, Bridgewater, NJ), and IVision software (BD Biosciences, Rockville, MD). The mercury light source was passed through a 360 ± 25 nm BP excitation filter, a 400 nm LP beam splitter, and a 410 nm LP emission filter. BMB was used as the standard nerve-specific fluorophore to which the fluorescence signal from all new fluorophores were compared. Images were collected with the following exposure times: 1mM = 5ms, 100 µM = 50ms, 10 µM = 125ms. Control nerve sections incubated with the IV formulation without fluorophore were also imaged. All subsequent fluorescence images were collected using the same exposure times as BMB, unless the fluorescence signal was saturated, in which case the integration time was reduced. Phase-contrast images of the same field of view were also collected. Fluorescence signal from each image was qualified using a 4-point scale: -, -/+, +, or + + (Table S5). The staining, imaging, and qualification were completed in triplicate.

All fluorophores with nerve fluorescence qualified as + or + + were purified using prep-HPLC and screened *ex vivo* in triplicate at 10 µM for nerve-specific fluorescence using the same procedure (Table S5). To validate the *ex vivo* screening assay for nerve-specificity, the fluorophores most structurally similar to previously published BMB (WH047_D2 – WH060_D15) and GE3082 (HW006_A6 – HW015_A15) [16] were also purified and further screened even if negative for nerve-specific fluorescence in the original crude product screen. Nerve-specificity was again compared with that of BMB stained nerve sections and qualified (Table S5). All images from the *ex vivo* nerve-specific assay of the purified compounds were quantified using region of interest analysis on three representative nerve-bundles, three representative lipid droplets, and three regions of background signal to calculate nerve to background and adipose to background fluorescence ratios.

### Quantitative Structure-Activity Relationship Modeling

The purified *ex vivo* nerve-specific screening data was used with the Discovery Studio 3.0 software package (Accelrys, Inc., San Diego, CA) for quantitative structure-activity relationship (QSAR) model generation. Three-dimensional structures for the 204 unique DSB analogs were generated from fingerprints followed by an energy minimization using the CHARM force field with default parameters. Bayesian categorization was used to create QSAR models and identify structural features that distinguish compounds with nerve-specific fluorescence (active) from compounds that did not show nerve-specific fluorescence (inactive). All compounds quantified as + and + + were classified as active, while all compounds quantified as - or -/+ were classified as inactive. Any crude compound quantified as - or -/+ and not further purified was also classified as inactive for model generation. The dataset of 204 unique molecules was randomly split into 80% for training and 20% for testing 5 times, where the testing portion of the dataset was not utilized during model development. For each random split, the “Create Bayesian Model” protocol was employed to train the model. The activity was predicted based on FCFP structural fingerprints where FCFP denotes the characteristics of the descriptors. The first 'F' represents the use of function classes as the atom abstraction method, where atoms are characterized as hydrogen-bond donors, hydrogen-bond acceptors, positively ionizable, negatively ionizable, etc., 'C' denotes that extended connectivity fingerprints were used, the second 'F' stands for fingerprints, and 'P' indicates that any duplicate features within a molecule are listed only once. Lastly, FCFP is denoted with a number, representing the maximum diameter in bond lengths of the largest structural feature used to generate the fingerprints. To test whether or not the quality of the QSAR model was influences by the feature size, models were built using FCFP6, FCFP8, and FCFP10 fingerprints. Besides testing the models on the external test sets (20% of the compounds), a 5-fold cross-validation was automatically performed on the training set (80% of the compounds).

### Animals

All animals used in this study were housed in an AAALAC-certified facility and studied under the supervision of an Institutional Animal Care and Use Committee (IACUC) protocol approved by the Beth Israel Deaconess Medical Center IACUC. CD-1 mice of either sex weighing 28 to 30 grams were purchased from Charles River Laboratories (Wilmington, MA) and used for all *in vivo* biodistribution studies. Female Yorkshire pigs weighing 30 kilograms were purchased from E. M. Parsons & Sons (Hadley, MA). Prior to surgery mice were anesthetized with a mixture of 100 mg/kg ketamine and 10 mg/kg xylazine (Webster Veterinary, Fort Devens, MA) administered intraperitoneally. Pig anesthesia was induced with a 4.4 mg/kg intramuscular Telazol (Fort Dodge Animal Health, For Dodge, IA) injection and maintained with 2% isoflurane after intubation. As stated in our IACUC approved rodent protocol, depth of rodent anesthesia was assessed through reactivity to toe pinch, where all animals were maintained under full anesthesia throughout the surgical procedures. All swine utilized in the current studies were kept under full anesthesia, with vital signs monitored, saline continuously delivered, and body temperature monitored and maintained

### Intraoperative Fluorescence Imaging Studies

The FLARE^TM^ intraoperative imaging system has been described in detail previously [28–31], and its use for nerve-specific imaging has been previously detailed in [16]. Images were collected at varied exposure times from 100–2000 ms. Images of vehicle injected control animals were also collected with corresponding exposure time. Color video images of the field of view were collected on a separate channel using custom-designed optics and software.

### In Vivo Biodistribution Studies

Purified compounds with positive *ex vivo* nerve-specific fluorescence were screened for nerve-specific fluorescence following systemic administration in CD-1 mice. Each compound was administered intravenously 4 hours prior to imaging (0.5 mg/mouse in 100 µL of the IV formulation). The 4-hour fluorophore-imaging interval was chosen from previous imaging studies [16]. The brachial plexus, sciatic, trigeminal ganglia, and optic nerves were dissected and imaged for each mouse. Each purified compound with positive *ex vivo* nerve-specific fluorescence was tested in a single mouse. The N/M and adipose to muscle (A/M) ratios of each dissected nerve site were calculated. The average and standard deviation of the N/M and A/M ratios for each nerve site was calculated for 5 vehicle-injected control animals. Any compound injected animal with N/M ratio greater than 1 standard deviation above the average N/M ratio of the vehicle injected control animals was tested in two additional mice to total n=3 for all compounds with positive *in vivo* nerve-specific fluorescence. The average and standard deviation of the N/M and A/M ratio for each brachial plexus (n=2 per animal), trigeminal ganglia (n=2 per animal), optic nerve (n=2 per animal), and sciatic nerve (n=1 dissected and image per animal) site was calculated for each compound.

To facilitate swine nerve imaging, dose-ranging studies were completed with the two new DSB fluorophores chosen for further study (WH159_K9 and HW099_G9). Mice were administered doses of 0.5, 0.25, and 0.125 mg/mouse for each compound. N/M ratio was calculated for the three doses of administered fluorophore. The 0.25 mg/mouse dose was chosen for scaling for swine studies, as N/M ratio was similar to that of the 0.5 mg/mouse dose.

### Ex Vivo Human Nerve-Specific Binding Studies

Human sciatic nerve tissue was harvested upon autopsy from a 40-year old female patient, 8 hours post mortem following an approved institutional review board (IRB) protocol. The Beth Israel Deaconess Medical Center IRB approved the protocol and determined that informed consent was not required as the acquired nerve tissues were considered discarded tissue. The tissue was washed with PBS and then flash frozen in OCT with liquid nitrogen. The same procedure as outline for the *ex vivo* nerve-specific fluorescence library screen was used to stain the human sciatic nerve tissue. All purified compounds positive for *ex vivo* pig nerve binding were tested on the human sciatic nerve at 100 µM and 10 µM fluorophore concentration in triplicate. The same fluorescence camera and filtration was used to image the nerve-specific fluorescence intensity from each DSB fluorophore. In addition, a QImaging 12-bit camera for color imaging (Surrey, BC, Canada) was used to capture the visible fluorescence emission from each of the nerve sections. Control human sciatic nerve sections that had been incubated with IV formulation not containing fluorophore were imaged using both the fluorescence and color cameras with the same integration time and normalization.

### Swine Nerve Imaging

The fluorophore dose for pig imaging was scaled from mouse studies by body surface area (BSA; 272-fold difference compared to mouse) for WH159_K9 and HW099_G9. Each fluorophore was administered at a BSA equivalent of 0.25 mg/mouse dose, or 68 mg in 30 ml of IV formulation, systemically 4 hours prior to imaging. Four pigs were used for the swine imaging study, where each pig received administration of one of the following: 68 mg WH159_K9, 68 mg HW099_G9, 68 mg BMB, or 30 ml IV formulation without fluorophore. Dissection of the brachial plexus nerve was completed 4 hours after fluorophore administration followed by imaging with the FLARE^TM^ system.

## Supporting Information

Figure S1 Average *in vivo* nerve-specific fluorescence of DSB fluorophores in n=3 mice. (TIFF)

Figure S2 *In vivo* nerve-specific fluorescence of DSB fluorophores with nerve fluorescence within 1 standard deviation of control autofluorescence for n=1 mouse. (TIFF)

Table S1 Compound number, IUPAC name, chemical formula, DSB chemical structure, right, middle, and left building block. (PDF)

Table S2 Chemical name of building block, CAS#, molecular weight, chemical formula, manufacturer, portion of molecule (right, middle, or left), building block type (bromo benzaldehyde, diethyl phosphonate, styrene), chemical structure. (PDF)

Table S3 Physiochemical properties of DSB library. (PDF)

Table S4 Spectral properties of DSB library. (PDF)

Table S5 *Ex vivo* nerve-specific screening assay results from DSB crude and purified library. (PDF)
