# Supplementary figures and images for: Structure-Activity Relationship of Nerve-Highlighting Fluorophores
Source: PLoS One. 2013 Sep 9;8(9):e73493. doi: 10.1371/journal.pone.0073493 (PMC3767781; doi:10.1371/journal.pone.0073493)

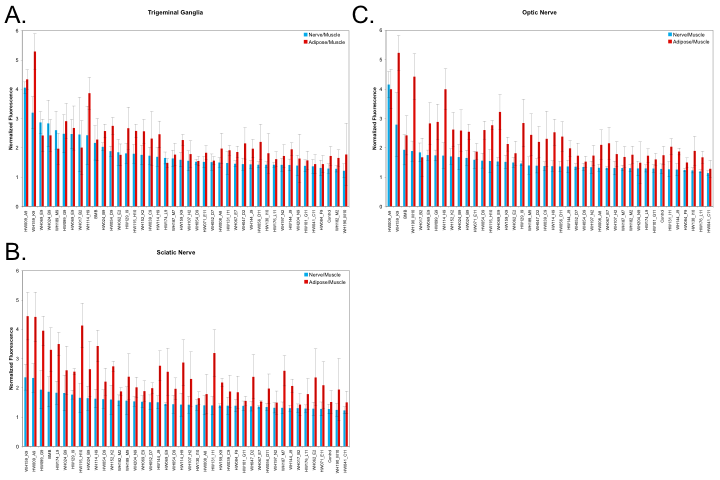

Supplement: Figure S1 — (TIFF) [file pone.0073493.s001.tiff]

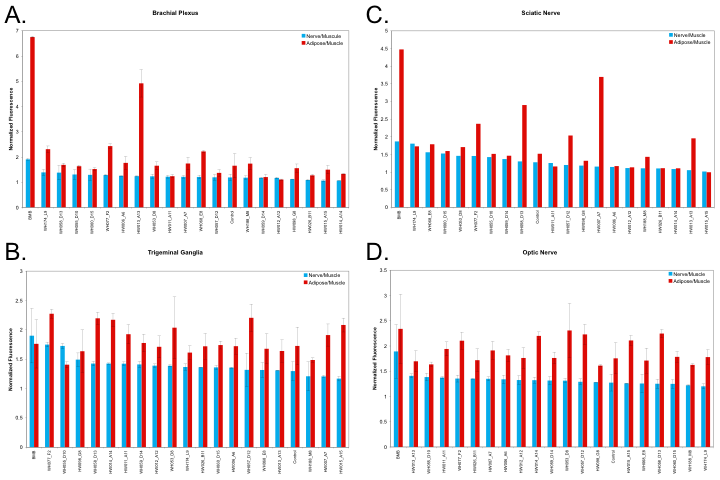

Supplement: Figure S2 — (TIFF) [file pone.0073493.s002.tiff]
