# Supplementary material for: Structure-Activity Relationship of Nerve-Highlighting Fluorophores
Source: PLoS One. 2013 Sep 9;8(9):e73493. doi: 10.1371/journal.pone.0073493 (PMC3767781; doi:10.1371/journal.pone.0073493)
Supplement: Table S1 — (PDF) [file pone.0073493.s003.pdf]

Table S1 - Chemical Structure

Compound #	IUPAC Name	Chemical Formula	*Structure*	*Head*	*Middle*	*Tail*
HW006_A6	4-(2-(4-aminostyryl)-4-methylstyryl)benzonitrile	$C_{24}H_{20}N_2$	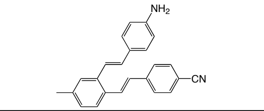	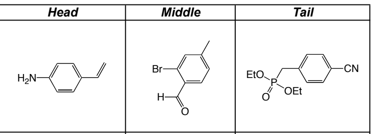		
HW007_A7	4-(2-(4-aminostyryl)-5-methoxystyryl)benzonitrile	$C_{24}H_{20}N_2O$	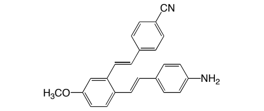	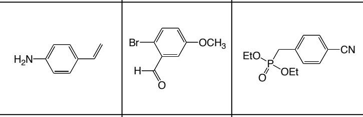		
HW008_A8	4-(3-(4-aminostyryl)-4-methoxystyryl)benzonitrile	$C_{24}H_{20}N_2O$	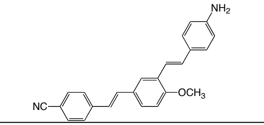	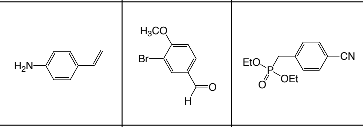		
HW009_A9	4-(4-(4-aminostyryl)-2-methoxystyryl)benzonitrile	$C_{24}H_{20}N_2O$	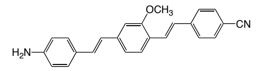	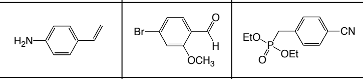		
HW010_A10	4-(5-(4-aminostyryl)-2-methoxystyryl)benzonitrile	$C_{24}H_{20}N_2O$	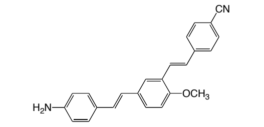	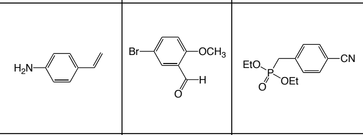		
HW011_A11	4-(5-(4-aminostyryl)-2-ethoxystyryl)benzonitrile	$C_{25}H_{22}N_2O$	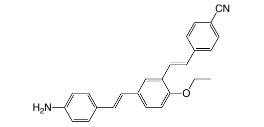	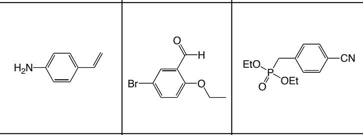		
HW012_A12	4-(5-(4-aminostyryl)-2,3-dimethoxystyryl)benzonitrile	$C_{25}H_{22}N_2O_2$	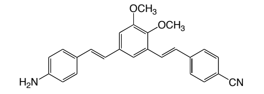	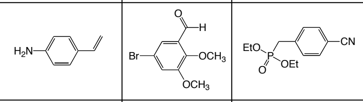		
HW013_A13	4-(5-(4-aminostyryl)-2,4-dimethoxystyryl)benzonitrile	$C_{25}H_{22}N_2O_2$	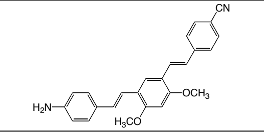	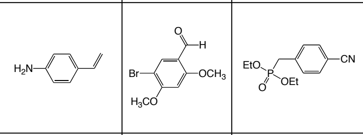		
HW014_A14	4-(3-(4-aminostyryl)-4,5-dimethoxystyryl)benzonitrile	$C_{25}H_{22}N_2O_2$	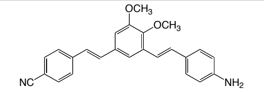	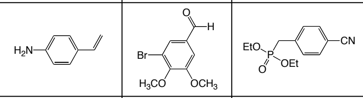		
HW015_A15	4-(2-(4-aminostyryl)-4,5-dimethoxystyryl)benzonitrile	$C_{25}H_{22}N_2O_2$	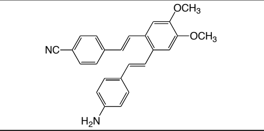	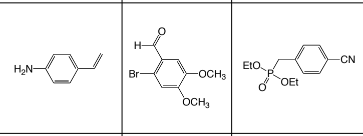		
HW021_B6	4-(2-(4-fluorostyryl)-5-methylstyryl)aniline	$C_{23}H_{20}FN$	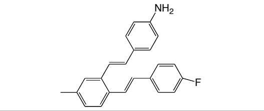	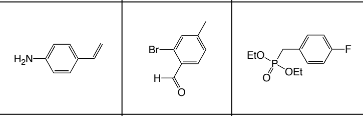		
HW022_B7	4-(2-(4-fluorostyryl)-4-methoxystyryl)aniline	$C_{23}H_{20}FNO$	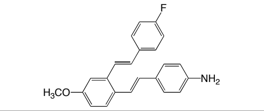	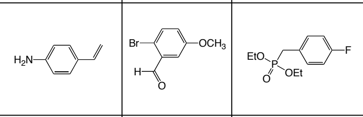		
HW023_B8	4-(5-(4-fluorostyryl)-2-methoxystyryl)aniline	$C_{23}H_{20}FNO$	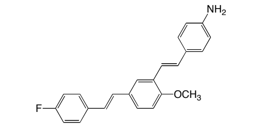	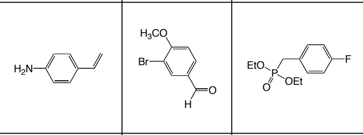		
HW024_B9	4-(4-(4-fluorostyryl)-3-methoxystyryl)aniline	$C_{23}H_{20}FNO$	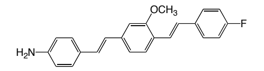	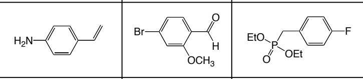		
HW025_B10	4-(3-(4-fluorostyryl)-4-methoxystyryl)aniline	$C_{23}H_{20}FNO$	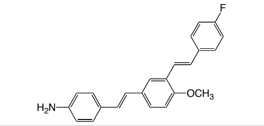	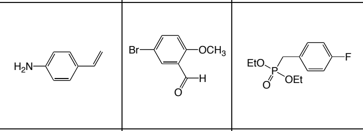		
HW026_B11	4-(4-ethoxy-3-(4-fluorostyryl)styryl)aniline	$C_{24}H_{22}FNO$	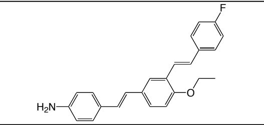	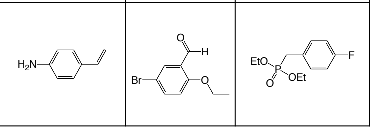		

HW027_B12	4-(3-(4-fluorostyryl)-4,5-dimethoxystyryl)aniline	$C_{24}H_{22}FNO_2$	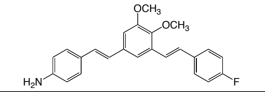	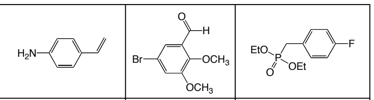		
HW028_B13	(E)-4-(5-(4-fluorophenethyl)-2,4-dimethoxystyryl)aniline	$C_{24}H_{24}FNO_2$	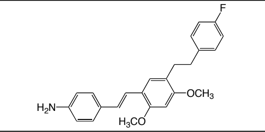	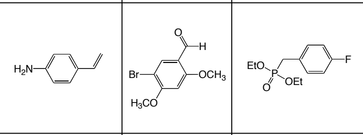		
HW029_B14	4-(5-(4-fluorostyryl)-2,3-dimethoxystyryl)aniline	$C_{24}H_{22}FNO_2$	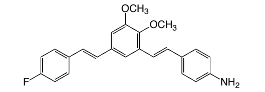	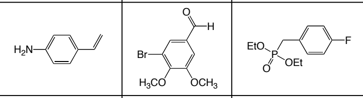		
HW030_B15	4-(2-(4-fluorostyryl)-4,5-dimethoxystyryl)aniline	$C_{24}H_{22}FNO_2$	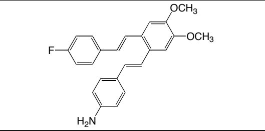	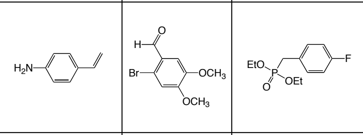		
HW036_C6	4-(2-(4-bromostyryl)-5-methylstyryl)aniline	$C_{23}H_{20}BrN$	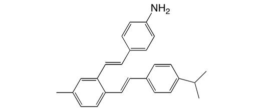	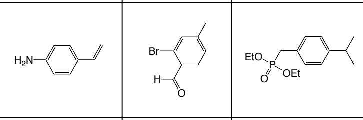		
HW037_C7	4-(2-(4-bromostyryl)-4-methoxystyryl)aniline	$C_{23}H_{20}BrNO$	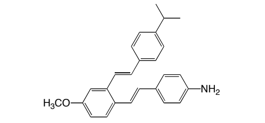	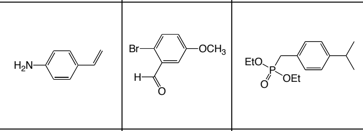		
HW038_C8	4-(5-(4-bromostyryl)-2-methoxystyryl)aniline	$C_{23}H_{20}BrNO$	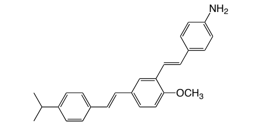	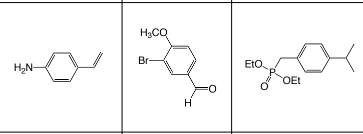		
HW039_C9	4-(4-(4-bromostyryl)-3-methoxystyryl)aniline	$C_{23}H_{20}BrNO$	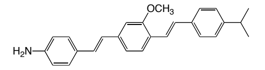	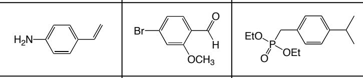		
HW040_C10	4-(3-(4-bromostyryl)-4-methoxystyryl)aniline	$C_{23}H_{20}BrNO$	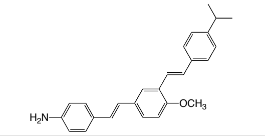	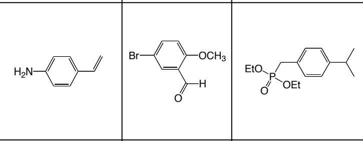		
HW041_C11	4-(3-(4-bromostyryl)-4-ethoxystyryl)aniline	$C_{24}H_{22}BrNO$	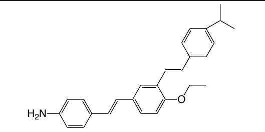	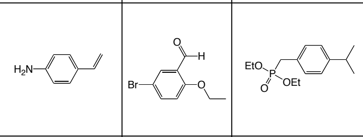		
HW042_C12	4-(3-(4-bromostyryl)-4,5-dimethoxystyryl)aniline	$C_{24}H_{22}BrNO_2$	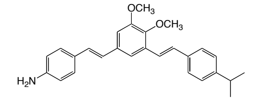	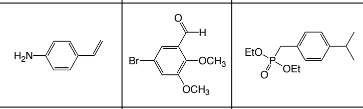		
HW043_C13	(E)-4-(5-(4-bromophenethyl)-2,4-dimethoxystyryl)aniline	$C_{24}H_{24}BrNO_2$	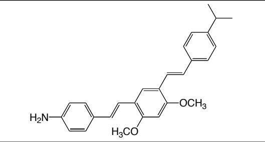	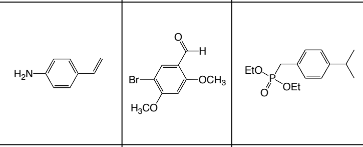		
HW044_C14	4-(5-(4-bromostyryl)-2,3-dimethoxystyryl)aniline	$C_{24}H_{22}BrNO_2$	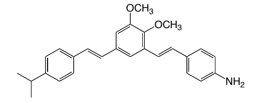	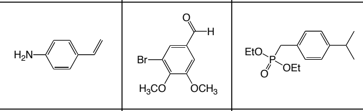		
HW045_C15	4-(2-(4-bromostyryl)-4,5-dimethoxystyryl)aniline	$C_{24}H_{22}BrNO_2$	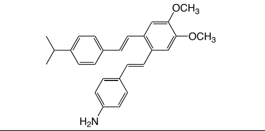	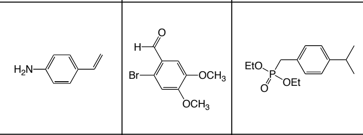		
HW051_D6	4-(2-(4-chlorostyryl)-5-methylstyryl)aniline	$C_{23}H_{20}ClN$	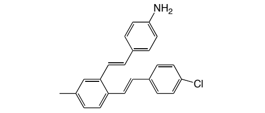	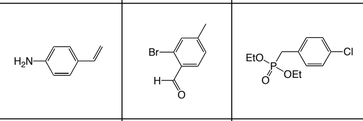		
HW052_D7	4-(2-(4-chlorostyryl)-4-methoxystyryl)aniline	$C_{23}H_{20}ClNO$	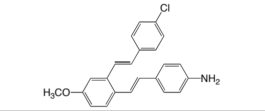	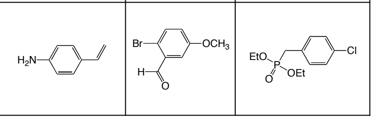		

HW053_D8	4-(5-(4-chlorostyryl)-2-methoxystyryl)aniline	$C_{23}H_{20}ClNO$	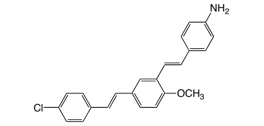	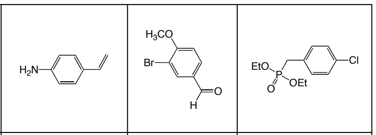		
HW054_D9	4-(4-(4-chlorostyryl)-3-methoxystyryl)aniline	$C_{23}H_{20}ClNO$	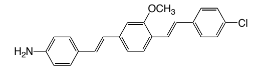	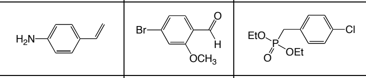		
HW055_D10	4-(3-(4-chlorostyryl)-4-methoxystyryl)aniline	$C_{23}H_{20}ClNO$	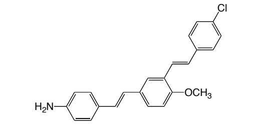	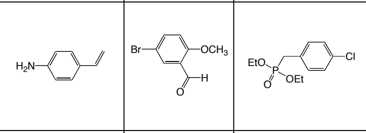		
HW056_D11	4-(3-(4-chlorostyryl)-4-ethoxystyryl)aniline	$C_{24}H_{22}ClNO$	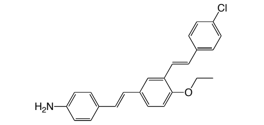	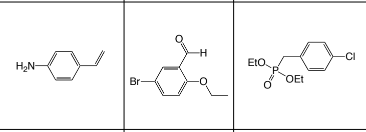		
HW057_D12	4-(3-(4-chlorostyryl)-4,5-dimethoxystyryl)aniline	$C_{24}H_{22}ClNO_2$	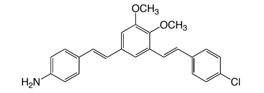	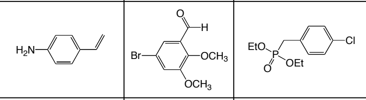		
HW058_D13	(E)-4-(5-(4-chlorophenethyl)-2,4-dimethoxystyryl)aniline	$C_{24}H_{24}ClNO_2$	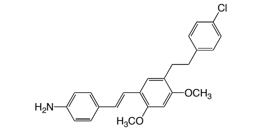	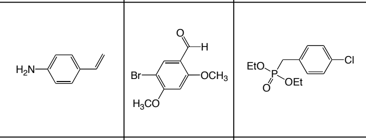		
HW059_D14	4-(5-(4-chlorostyryl)-2,3-dimethoxystyryl)aniline	$C_{24}H_{22}ClNO_2$	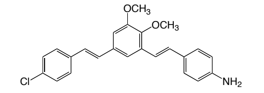	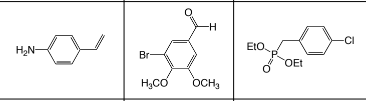		
HW060_D15	4-(2-(4-chlorostyryl)-4,5-dimethoxystyryl)aniline	$C_{24}H_{22}ClNO_2$	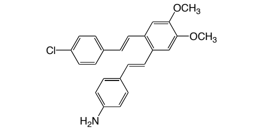	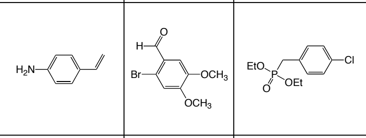		
HW066_E6	4-(2-(4-bromostyryl)-5-methylstyryl)aniline	$C_{23}H_{20}BrN$	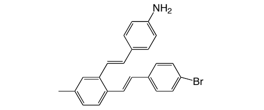	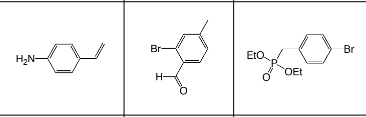		
HW067_E7	4-(2-(4-bromostyryl)-4-methoxystyryl)aniline	$C_{23}H_{20}BrNO$	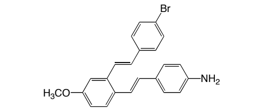	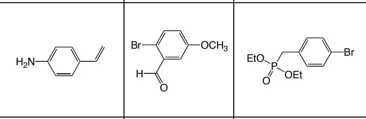		
HW068_E8	4-(5-(4-bromostyryl)-2-methoxystyryl)aniline	$C_{23}H_{20}BrNO$	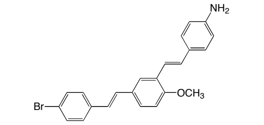	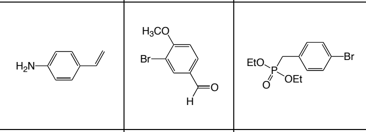		
HW069_E9	4-(4-(4-bromostyryl)-3-methoxystyryl)aniline	$C_{23}H_{20}BrNO$	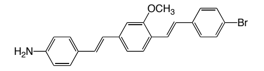	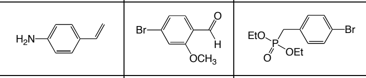		
HW070_E10	4-(3-(4-bromostyryl)-4-methoxystyryl)aniline	$C_{23}H_{20}BrNO$	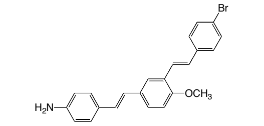	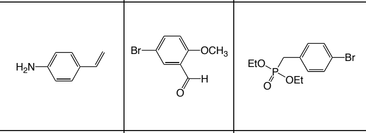		
HW071_E11	4-(3-(4-bromostyryl)-4-ethoxystyryl)aniline	$C_{24}H_{22}BrNO$	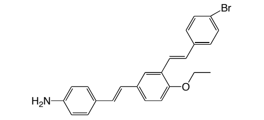	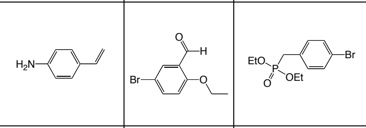		
HW072_E12	4-(3-(4-bromostyryl)-4,5-dimethoxystyryl)aniline	$C_{24}H_{22}BrNO_2$	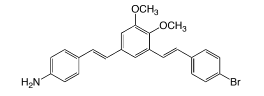	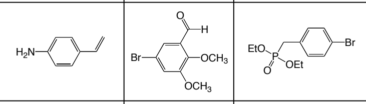		
HW073_E13	(E)-4-(5-(4-bromophenethyl)-2,4-dimethoxystyryl)aniline	$C_{24}H_{24}BrNO_2$	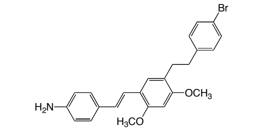	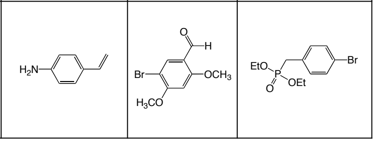		

HW074_E14	4-(5-(4-bromostyryl)-2,3-dimethoxystyryl)aniline	$C_{24}H_{22}BrNO_2$	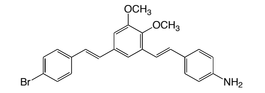	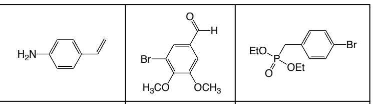		
HW075_E15	4-(2-(4-bromostyryl)-4,5-dimethoxystyryl)aniline	$C_{24}H_{22}BrNO_2$	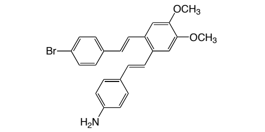	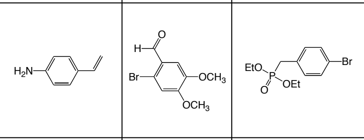		
HW081_F6	4-(2-(4-iodostyryl)-5-methylstyryl)aniline	$C_{23}H_{20}IN$	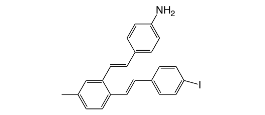	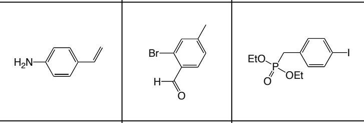		
HW082_F7	4-(2-(4-iodostyryl)-5-methylstyryl)aniline	$C_{23}H_{20}INO$	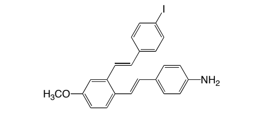	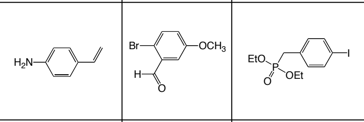		
HW083_F8	4-(2-(4-iodostyryl)-5-methylstyryl)aniline	$C_{23}H_{20}INO$	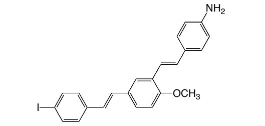	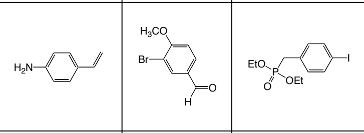		
HW084_F9	4-(4-(4-iodostyryl)-3-methoxystyryl)aniline	$C_{23}H_{20}INO$	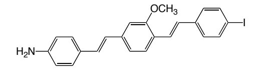	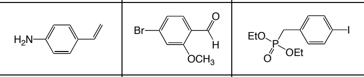		
HW085_F10	4-(3-(4-iodostyryl)-4-methoxystyryl)aniline	$C_{23}H_{20}INO$	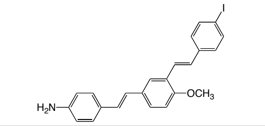	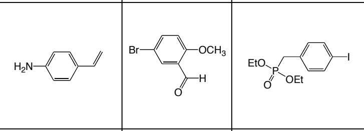		
HW086_F11	4-(4-ethoxy-3-(4-iodostyryl)styryl)aniline	$C_{24}H_{22}INO$	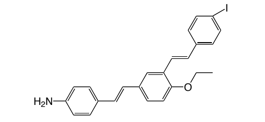	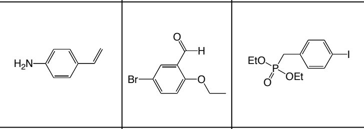		
HW087_F12	4-(3-(4-iodostyryl)-4,5-dimethoxystyryl)aniline	$C_{24}H_{22}INO_2$	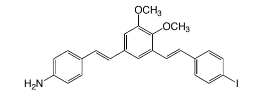	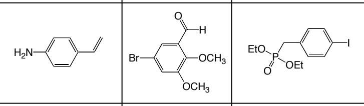		
HW088_F13	4-(5-(4-iodostyryl)-2,4-dimethoxystyryl)aniline	$C_{24}H_{22}INO_2$	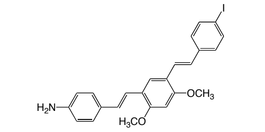	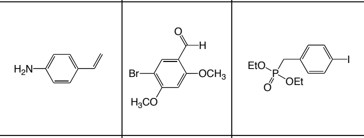		
HW089_F14	4-(5-(4-iodostyryl)-2,3-dimethoxystyryl)aniline	$C_{24}H_{22}INO_2$	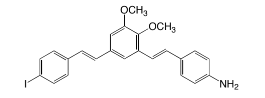	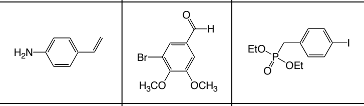		
HW090_F15	4-(2-(4-iodostyryl)-4,5-dimethoxystyryl)aniline	$C_{24}H_{22}INO_2$	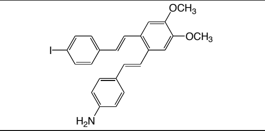	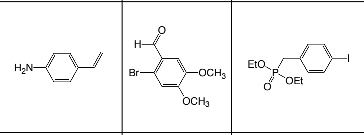		
HW096_G6	4-(2-(4-methoxystyryl)-5-methylstyryl)aniline	$C_{24}H_{23}NO$	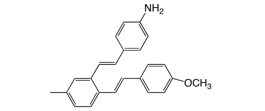	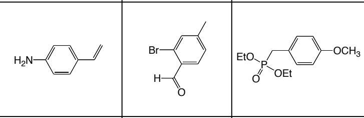		
HW097_G7	4-(4-methoxy-2-(4-methoxystyryl)styryl)aniline	$C_{24}H_{23}NO_2$	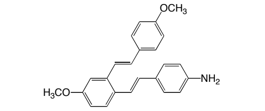	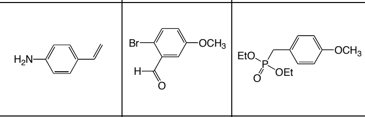		
HW098_G8	4-(2-methoxy-5-(4-methoxystyryl)styryl)aniline	$C_{24}H_{23}NO_2$	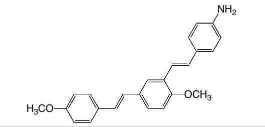	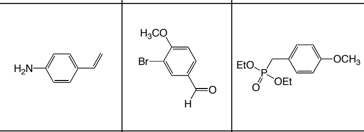		
HW099_G9	4-(3-methoxy-4-(4-methoxystyryl)styryl)aniline	$C_{24}H_{23}NO_2$	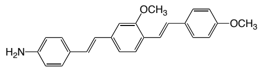	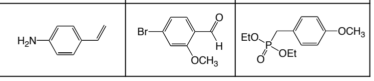		

HW100_G10	4-(4-methoxy-3-(4-methoxystyryl)styryl)aniline	$C_{24}H_{23}NO_2$	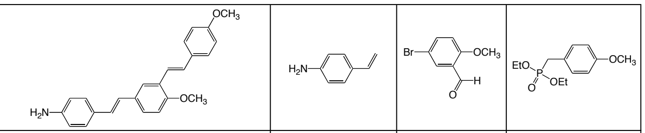			
HW101_G11	4-(4-ethoxy-3-(4-methoxystyryl)styryl)aniline	$C_{25}H_{25}NO_2$	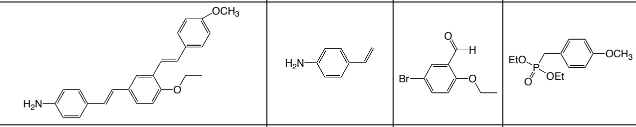			
HW102_G12	4-(3,4-dimethoxy-5-(4-methoxystyryl)styryl)aniline	$C_{25}H_{25}NO_3$	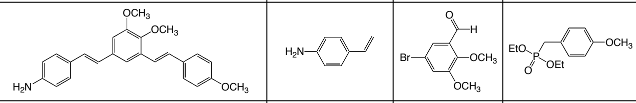			
HW103_G13	4-(2,4-dimethoxy-5-(4-methoxystyryl)styryl)aniline	$C_{25}H_{25}NO_3$	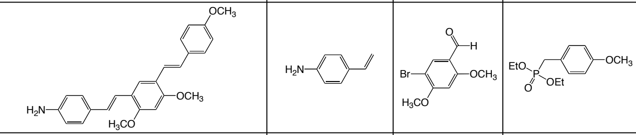			
HW104_G14	4-(2,3-dimethoxy-5-(4-methoxystyryl)styryl)aniline	$C_{25}H_{25}NO_3$	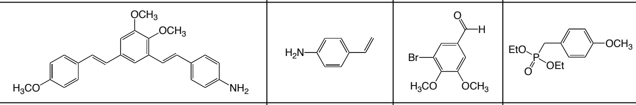			
HW105_G15	4-(4,5-dimethoxy-2-(4-methoxystyryl)styryl)aniline	$C_{25}H_{25}NO_3$	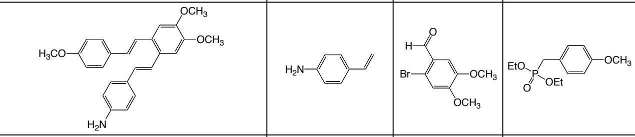			
HW111_H6	4-(5-methyl-2-(4-methylstyryl)styryl)aniline	$C_{24}H_{23}N$	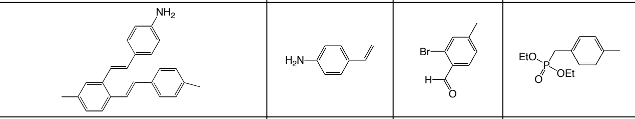			
HW112_H7	4-(4-methoxy-2-(4-methylstyryl)styryl)aniline	$C_{24}H_{23}N$	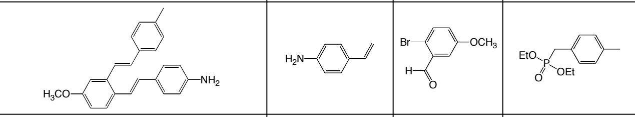			
HW113_H8	4-(2-methoxy-5-(4-methylstyryl)styryl)aniline	$C_{24}H_{23}NO$	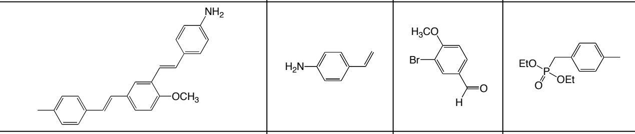			
HW114_H9	4-(3-methoxy-4-(4-methylstyryl)styryl)aniline	$C_{24}H_{23}NO$	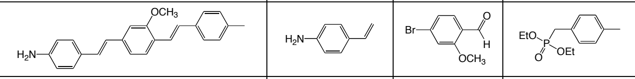			
HW115_H10	4-(4-methoxy-3-(4-methylstyryl)styryl)aniline	$C_{24}H_{23}NO$	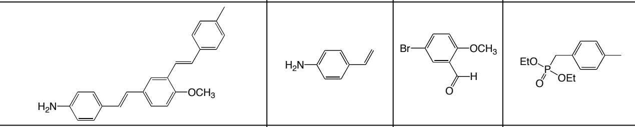			
HW116_H11	4-(4-ethoxy-3-(4-methylstyryl)styryl)aniline	$C_{25}H_{25}NO$	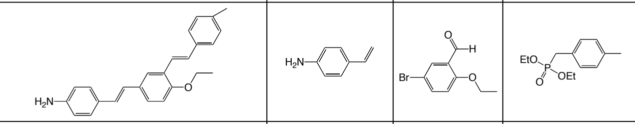			
HW117_H12	4-(3,4-dimethoxy-5-(4-methylstyryl)styryl)aniline	$C_{25}H_{25}NO_2$	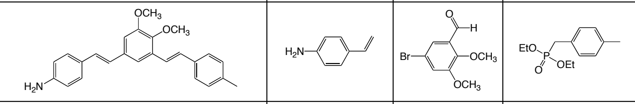			
HW118_H13	4-(2,4-dimethoxy-5-(4-methylstyryl)styryl)aniline	$C_{25}H_{25}NO_2$	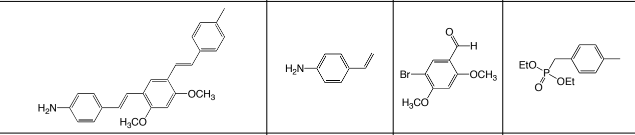			
HW119_H14	4-(2,3-dimethoxy-5-(4-methylstyryl)styryl)aniline	$C_{25}H_{25}NO_2$	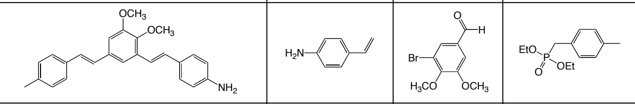			
HW120_H15	4-(4,5-dimethoxy-2-(4-methylstyryl)styryl)aniline	$C_{25}H_{25}NO_2$	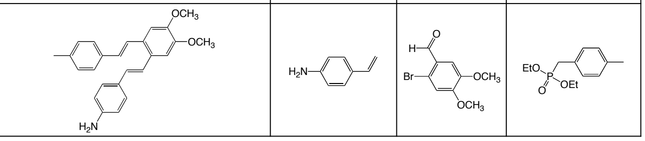			

HW126_I6	4-(5-methyl-2-(2-methylstyryl)styryl)aniline	$C_{24}H_{23}N$	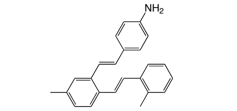	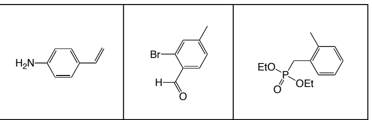		
HW127_I7	4-(4-methoxy-2-(2-methylstyryl)styryl)aniline	$C_{24}H_{23}NO$	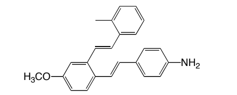	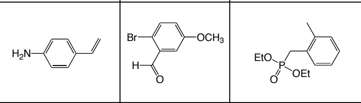		
HW128_I8	4-(2-methoxy-5-(2-methylstyryl)styryl)aniline	$C_{24}H_{23}NO$	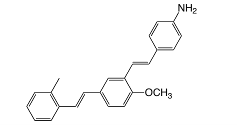	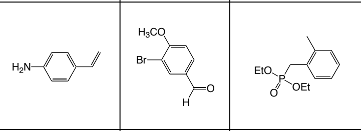		
HW129_I9	4-(3-methoxy-4-(2-methylstyryl)styryl)aniline	$C_{24}H_{23}NO$	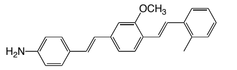	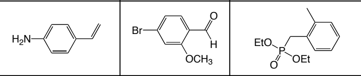		
HW130_I10	4-(4-methoxy-3-(2-methylstyryl)styryl)aniline	$C_{24}H_{23}NO$	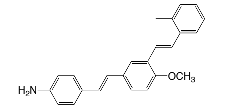	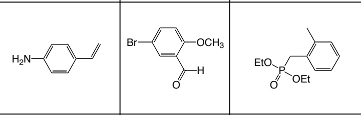		
HW131_I11	4-(4-ethoxy-3-(2-methylstyryl)styryl)aniline	$C_{25}H_{25}NO$	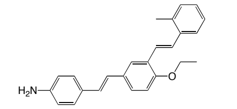	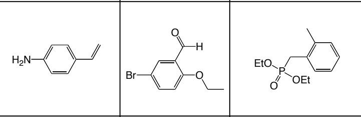		
HW132_I12	4-(3,4-dimethoxy-5-(2-methylstyryl)styryl)aniline	$C_{25}H_{25}NO_2$	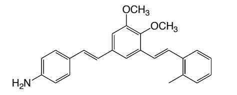	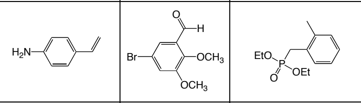		
HW133_I13	4-(2,4-dimethoxy-5-(2-methylstyryl)styryl)aniline	$C_{25}H_{25}NO_2$	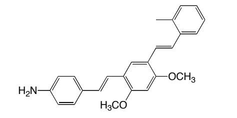	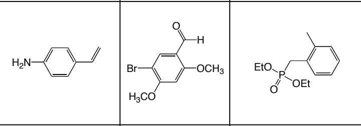		
HW134_I14	4-(2,3-dimethoxy-5-(2-methylstyryl)styryl)aniline	$C_{25}H_{25}NO_2$	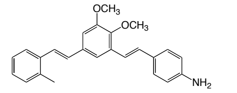	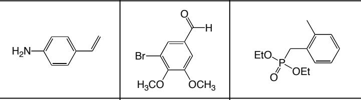		
HW135_I15	4-(4,5-dimethoxy-2-(2-methylstyryl)styryl)aniline	$C_{25}H_{25}NO_2$	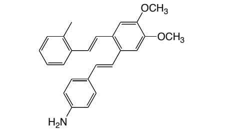	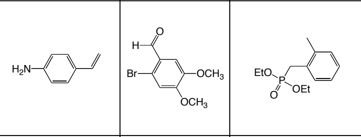		
HW141_J6	4-(5-methyl-2-(3-methylstyryl)styryl)aniline	$C_{24}H_{23}N$	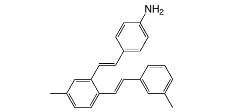	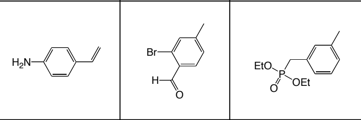		
HW142_J7	4-(4-methoxy-2-(3-methylstyryl)styryl)aniline	$C_{24}H_{23}NO$	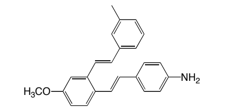	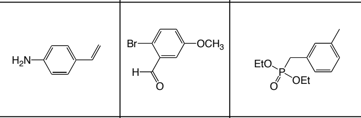		
HW143_J8	4-(2-methoxy-5-(3-methylstyryl)styryl)aniline	$C_{24}H_{23}NO$	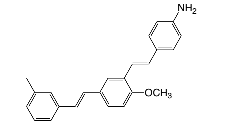	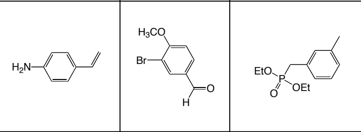		
HW144_J9	4-(3-methoxy-4-(3-methylstyryl)styryl)aniline	$C_{24}H_{23}NO$	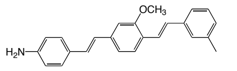	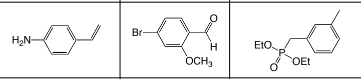		
HW145_J10	4-(4-methoxy-3-(3-methylstyryl)styryl)aniline	$C_{24}H_{23}NO$	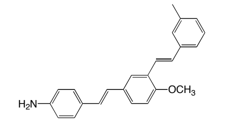	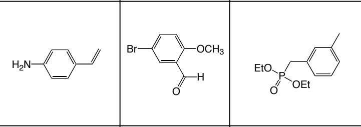		
HW146_J11	4-(4-ethoxy-3-(3-methylstyryl)styryl)aniline	$C_{25}H_{25}NO$	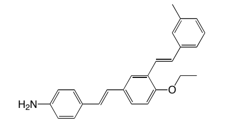	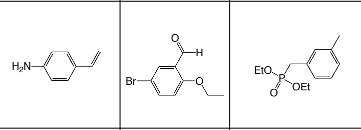		
HW147_J12	4-(3,4-dimethoxy-5-(3-methylstyryl)styryl)aniline	$C_{25}H_{25}NO_2$	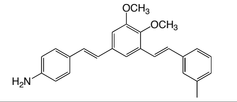	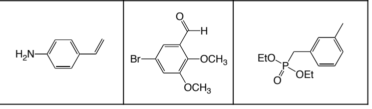		

HW148_J13	4-(2,4-dimethoxy-5-(3-methylstyryl)styryl)aniline	$C_{25}H_{25}NO_2$	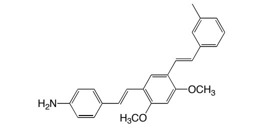	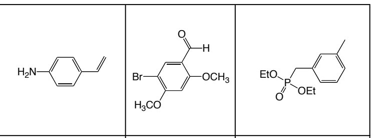
HW149_J14	4-(2,3-dimethoxy-5-(3-methylstyryl)styryl)aniline	$C_{25}H_{25}NO_2$	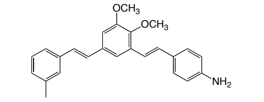	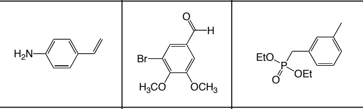
HW150_J15	4-(4,5-dimethoxy-2-(3-methylstyryl)styryl)aniline	$C_{25}H_{25}NO_2$	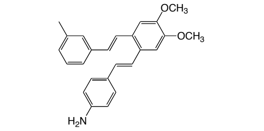	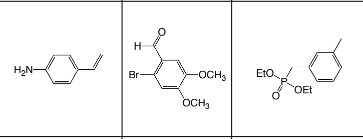
HW156_K6	4,4'-(1E,1'E)-2,2'-(4-methyl-1,2-phenylene)bis(ethene-2,1-diyl)dianiline	$C_{23}H_{22}N_2$	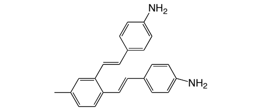	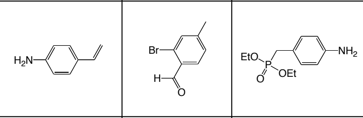
HW157_K7	4,4'-(1E,1'E)-2,2'-(4-methoxy-1,2-phenylene)bis(ethene-2,1-diyl)dianiline	$C_{23}H_{22}N_2O$	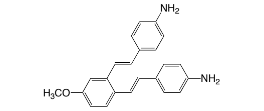	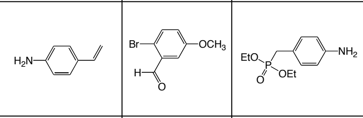
HW158_K8	4,4'-(1E,1'E)-2,2'-(4-methoxy-1,3-phenylene)bis(ethene-2,1-diyl)dianiline	$C_{23}H_{22}N_2O$	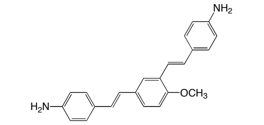	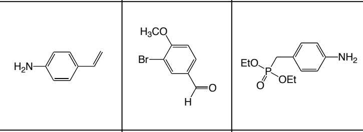
HW159_K9	4,4'-(1E,1'E)-2,2'-(2-methoxy-1,4-phenylene)bis(ethene-2,1-diyl)dianiline	$C_{23}H_{22}N_2O$	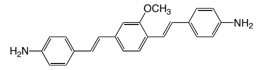	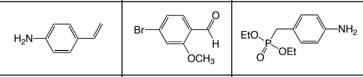
HW160_K10	4,4'-(1E,1'E)-2,2'-(4-methoxy-1,3-phenylene)bis(ethene-2,1-diyl)dianiline	$C_{23}H_{22}N_2O$	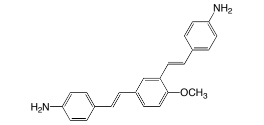	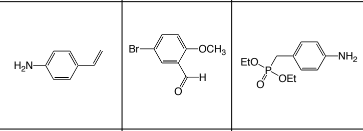
HW161_K11	4,4'-(1E,1'E)-2,2'-(4-ethoxy-1,3-phenylene)bis(ethene-2,1-diyl)dianiline	$C_{24}H_{24}N_2O$	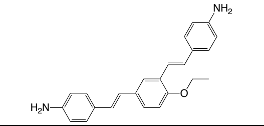	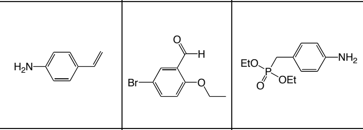
HW162_K12	4,4'-(1E,1'E)-2,2'-(4,5-dimethoxy-1,3-phenylene)bis(ethene-2,1-diyl)dianiline	$C_{24}H_{24}N_2O_2$	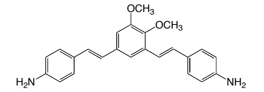	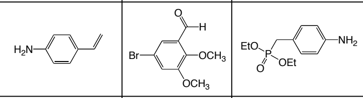
HW163_K13	4,4'-(1E,1'E)-2,2'-(4,6-dimethoxy-1,3-phenylene)bis(ethene-2,1-diyl)dianiline	$C_{24}H_{24}N_2O_2$	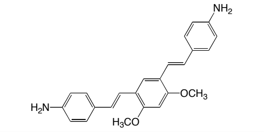	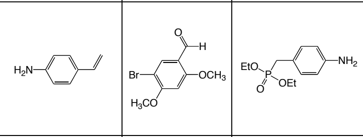
HW164_K14	4,4'-(1E,1'E)-2,2'-(4,5-dimethoxy-1,3-phenylene)bis(ethene-2,1-diyl)dianiline	$C_{24}H_{24}N_2O_2$	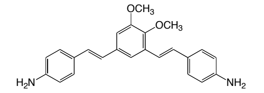	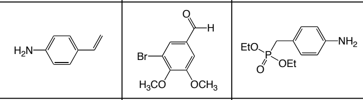
HW165_K15	4,4'-(1E,1'E)-2,2'-(4,5-dimethoxy-1,2-phenylene)bis(ethene-2,1-diyl)dianiline	$C_{24}H_{24}N_2O_2$	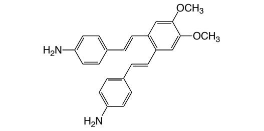	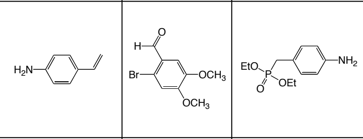
HW171_L6	4-(2-(3-chlorostyryl)-5-methylstyryl)aniline	$C_{23}H_{20}ClN$	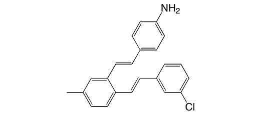	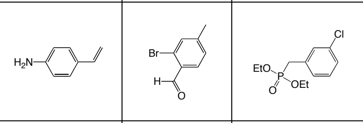
HW172_L7	4-(2-(3-chlorostyryl)-4-methoxystyryl)aniline	$C_{23}H_{20}ClNO$	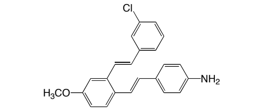	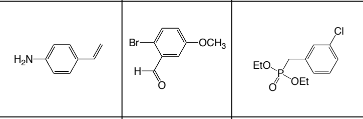
HW173_L8	4-(5-(3-chlorostyryl)-2-methoxystyryl)aniline	$C_{23}H_{20}ClNO$	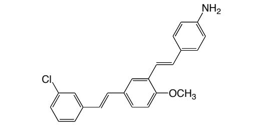	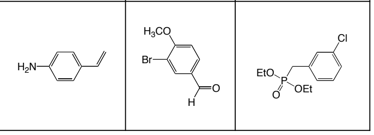

HW174_L9	4-(4-(3-chlorostyryl)-3-methoxystyryl)aniline	$C_{23}H_{20}ClNO$	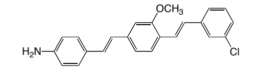	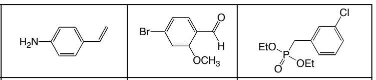		
HW175_L10	4-(3-(3-chlorostyryl)-4-methoxystyryl)aniline	$C_{23}H_{20}ClNO$	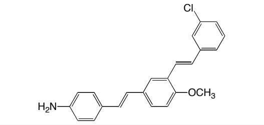	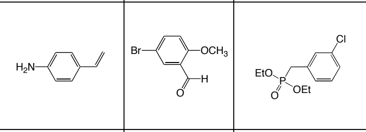		
HW176_L11	4-(3-(3-chlorostyryl)-4-ethoxystyryl)aniline	$C_{23}H_{20}ClNO$	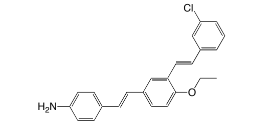	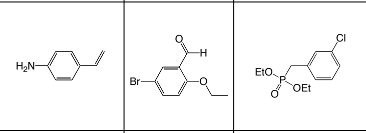		
HW177_L12	4-(3-(3-chlorostyryl)-4,5-dimethoxystyryl)aniline	$C_{23}H_{20}ClNO_2$	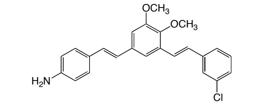	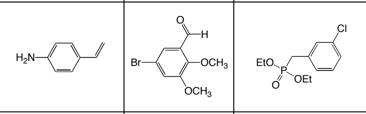		
HW178_L13	4-(5-(3-chlorostyryl)-2,4-dimethoxystyryl)aniline	$C_{23}H_{20}ClNO_2$	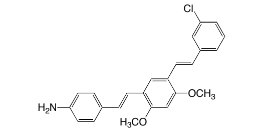	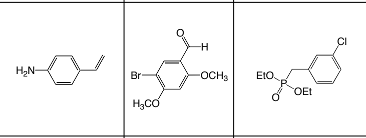		
HW179_L14	4-(5-(3-chlorostyryl)-2,3-dimethoxystyryl)aniline	$C_{23}H_{20}ClNO_2$	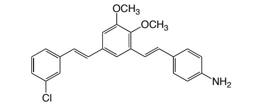	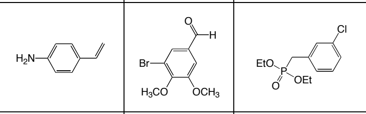		
HW180_L15	4-(2-(3-chlorostyryl)-4,5-dimethoxystyryl)aniline	$C_{23}H_{20}ClNO_2$	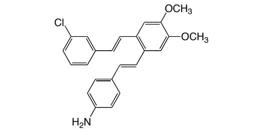	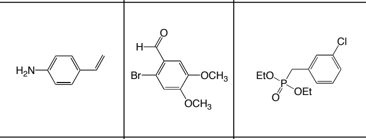		
WH017_B2	4-(4-(2,4-dimethylstyryl)-3-fluorostyryl)aniline	C24H22FN	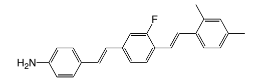	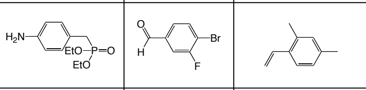		
WH020_B5	4-((E)-2-(5-(2,4-dimethylstyryl)benzo[d][1,3]dioxol-4-yl)vinyl)aniline	$C_{25}H_{23}NO_2$	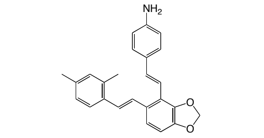	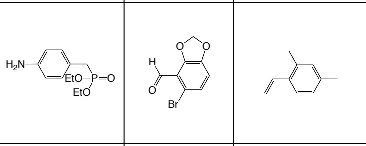		
WH021_B6	4-(2-(2,4-dimethylstyryl)-4-methylstyryl)aniline	$C_{25}H_{25}N$	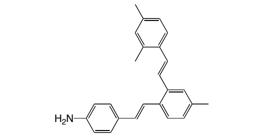	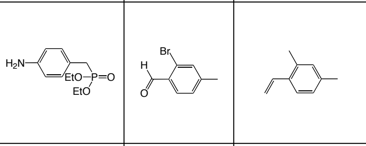		
WH022_B7	4-(2-(3,5-dimethylstyryl)-5-methoxystyryl)aniline	$C_{25}H_{25}NO$	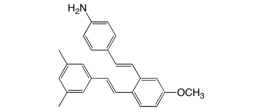	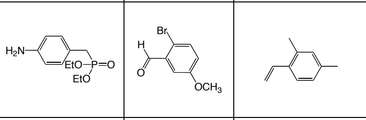		
WH023_B8	4-(3-(2,4-dimethylstyryl)-4-methoxystyryl)aniline	$C_{25}H_{25}NO$	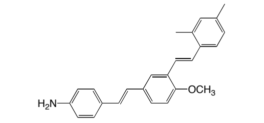	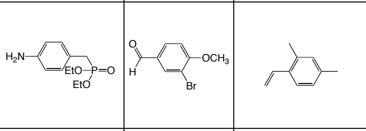		
WH024_B9	4-(4-(2,4-dimethylstyryl)-2-methoxystyryl)aniline	$C_{25}H_{25}NO$	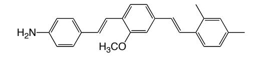	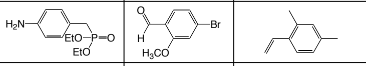		
WH025_B10	4-(5-(2,4-dimethylstyryl)-2-methoxystyryl)aniline	$C_{25}H_{25}NO$	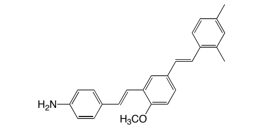	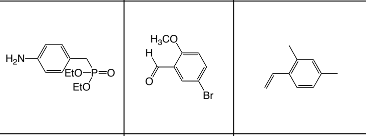		
WH027_B12	4-(5-(2,4-dimethylstyryl)-2,3-dimethoxystyryl)aniline	$C_{26}H_{27}NO_2$	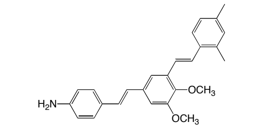	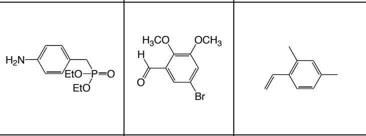		
WH028_B13	4-(5-(2,4-dimethylstyryl)-2,4-dimethoxystyryl)aniline	$C_{26}H_{27}NO_2$	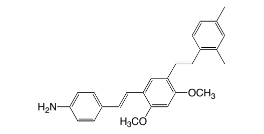	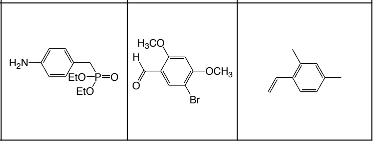		

WH029_B14	4-(3-(2,4-dimethylstyryl)-4,5-dimethoxystyryl)aniline	$C_{26}H_{27}NO_2$	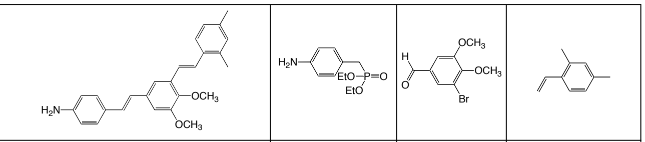
WH030_B15	4-(2-(2,4-dimethylstyryl)-4,5-dimethoxystyryl)aniline	$C_{26}H_{27}NO_2$	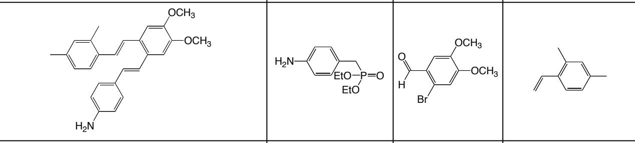
WH047_D2	4,4'-(1E,1'E)-2,2'-(2-fluoro-1,4-phenylene)bis(ethene-2,1-diyl)dianiline	$C_{22}H_{19}FN_2$	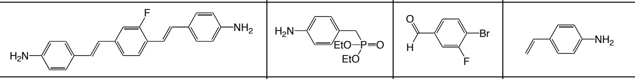
WH050_D5	4,4'-(1E,1'E)-2,2'-(benzo[d][1,3]dioxole-4,5-diyl)bis(ethene-2,1-diyl)dianiline	$C_{23}H_{20}N_2O_2$	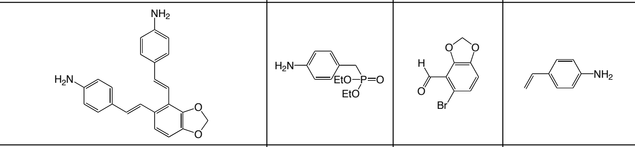
WH051_D6	(E)-4-(2-(4-aminophenethyl)-4-methylstyryl)aniline	$C_{23}H_{22}N_2$	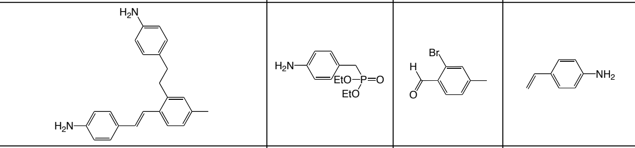
WH052_D7	4,4'-(1E,1'E)-2,2'-(4-methoxy-1,2-phenylene)bis(ethene-2,1-diyl)dianiline	$C_{23}H_{22}N_2O$	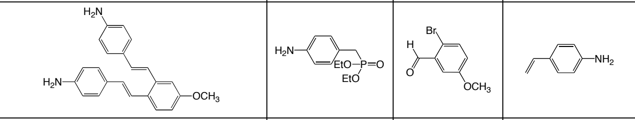
WH053_D8	4,4'-(1E,1'E)-2,2'-(4-methoxy-1,3-phenylene)bis(ethene-2,1-diyl)dianiline	$C_{23}H_{22}N_2O$	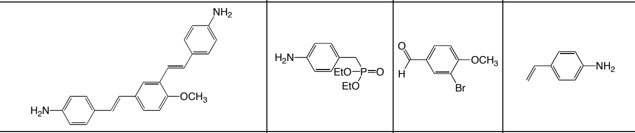
WH054_D9	4,4'-(1E,1'E)-2,2'-(2-methoxy-1,4-phenylene)bis(ethene-2,1-diyl)dianiline	$C_{23}H_{22}N_2O$	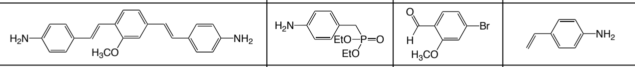
WH055_D10	4,4'-(1E,1'E)-2,2'-(4-methoxy-1,3-phenylene)bis(ethene-2,1-diyl)dianiline	$C_{23}H_{22}N_2O$	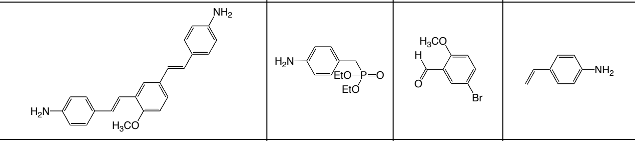
WH057_D12	4,4'-(1E,1'E)-2,2'-(4,5-dimethoxy-1,3-phenylene)bis(ethene-2,1-diyl)dianiline	$C_{24}H_{24}N_2O_2$	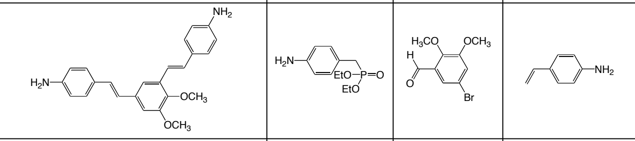
WH058_D13	4,4'-(1E,1'E)-2,2'-(4,6-dimethoxy-1,3-phenylene)bis(ethene-2,1-diyl)dianiline	$C_{24}H_{24}N_2O_2$	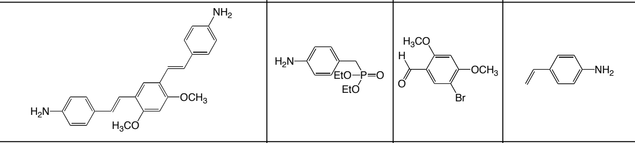
WH059_D14	4,4'-(1E,1'E)-2,2'-(4,5-dimethoxy-1,3-phenylene)bis(ethene-2,1-diyl)dianiline	$C_{24}H_{24}N_2O_2$	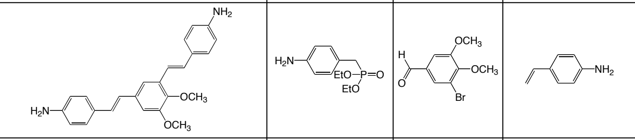
WH060_D15	4,4'-(1E,1'E)-2,2'-(4,5-dimethoxy-1,2-phenylene)bis(ethene-2,1-diyl)dianiline	$C_{24}H_{24}N_2O_2$	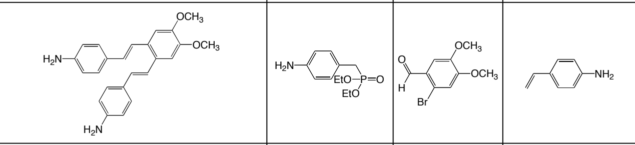
WH062_E2	4-(3-fluoro-4-((E)-2-(naphthalen-2-yl)vinyl)styryl)aniline	$C_{26}H_{20}FN$	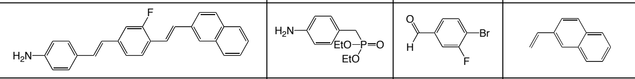
WH065_E5	4-((E)-2-(5-((E)-2-(naphthalen-2-yl)vinyl)benzo[d][1,3]dioxol-4-yl)vinyl)aniline	$C_{27}H_{21}NO_2$	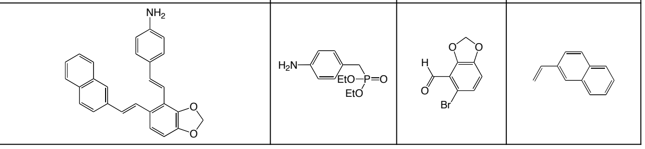

WH066_E6	4-(4-methyl-2-((E)-2-(naphthalen-1-yl)vinyl)styryl)aniline	$C_{27}H_{23}NO$	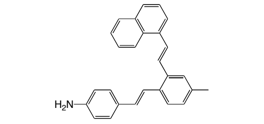	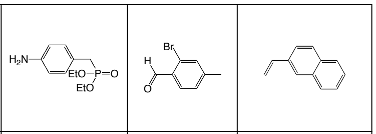
WH067_E7	4-(5-methoxy-2-((E)-2-(naphthalen-2-yl)vinyl)styryl)aniline	$C_{27}H_{23}N$	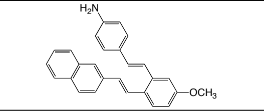	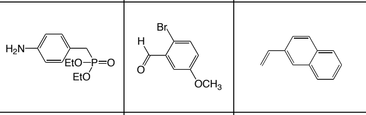
WH068_E8	4-(4-methoxy-3-((E)-2-(naphthalen-2-yl)vinyl)styryl)aniline	$C_{27}H_{23}NO$	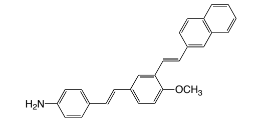	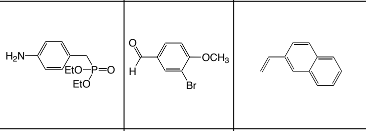
WH069_E9	4-(2-methoxy-4-((E)-2-(naphthalen-2-yl)vinyl)styryl)aniline	$C_{27}H_{23}NO$	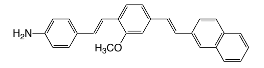	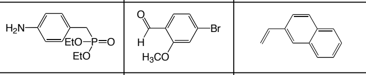
WH070_E10	4-(2-methoxy-5-((E)-2-(naphthalen-1-yl)vinyl)styryl)aniline	$C_{27}H_{23}NO$	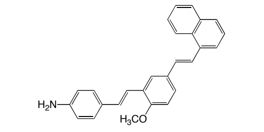	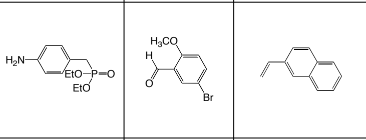
WH072_E12	4-(2,3-dimethoxy-5-((E)-2-(naphthalen-2-yl)vinyl)styryl)aniline	$C_{28}H_{25}NO_2$	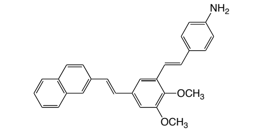	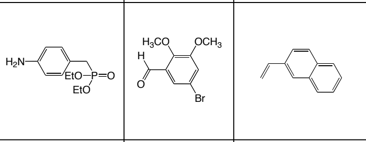
WH073_E13	4-(2,4-dimethoxy-5-((E)-2-(naphthalen-1-yl)vinyl)styryl)aniline	$C_{28}H_{25}NO_2$	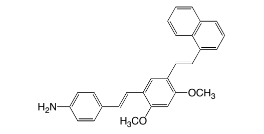	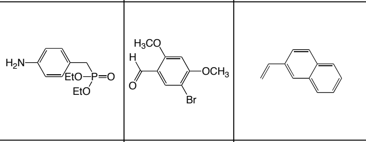
WH074_E14	4-(3,4-dimethoxy-5-((E)-2-(naphthalen-1-yl)vinyl)styryl)aniline	$C_{28}H_{25}NO_2$	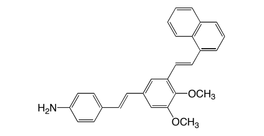	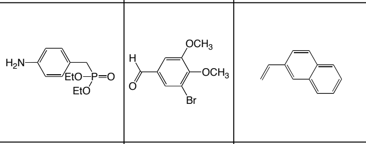
WH075_E15	4-(4,5-dimethoxy-2-((E)-2-(naphthalen-1-yl)vinyl)styryl)aniline	$C_{28}H_{25}NO_2$	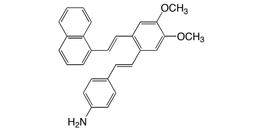	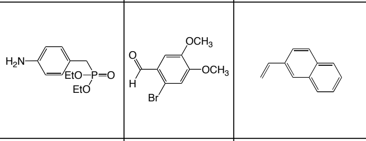
WH077_F2	4-(4-(3,4-dimethoxystyryl)-3-fluorostyryl)aniline	$C_{24}H_{22}FNO_2$	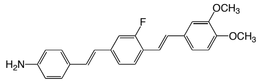	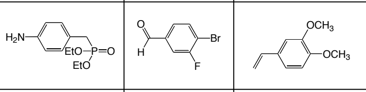
WH080_F5	4-((E)-2-(5-(3,4-dimethoxystyryl)benzo[d][1,3]dioxol-4-yl)vinyl)aniline	$C_{25}H_{23}NO_4$	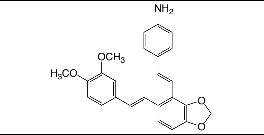	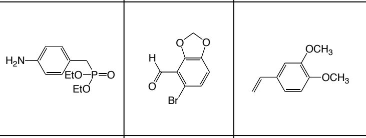
WH081_F6	4-(2-(3,4-dimethoxystyryl)-4-methylstyryl)aniline	$C_{25}H_{25}NO_2$	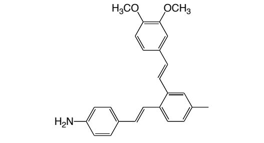	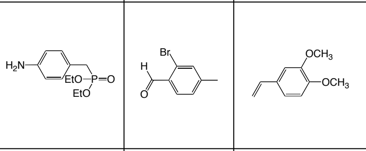
WH082_F7	4-(2-(3,4-dimethoxystyryl)-5-methoxystyryl)aniline	$C_{25}H_{25}NO_3$	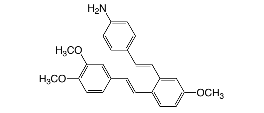	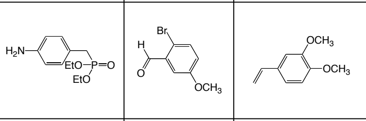
WH083_F8	4-(3-(3,4-dimethoxystyryl)-4-methoxystyryl)aniline	$C_{25}H_{25}NO_3$	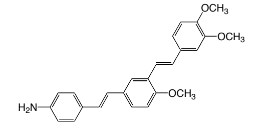	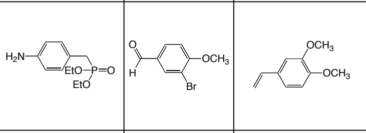
WH084_F9	4-(4-(3,4-dimethoxystyryl)-2-methoxystyryl)aniline	$C_{25}H_{25}NO_3$	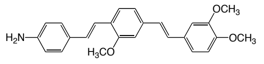	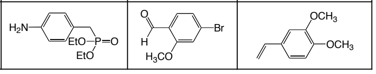

WH085_F10	4-(5-(3,4-dimethoxystyryl)-2-methoxystyryl)aniline	$C_{25}H_{25}NO_3$	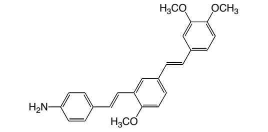	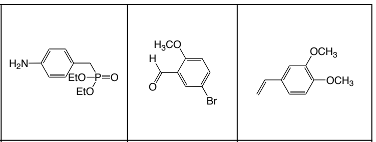		
WH087_F12	4-(5-(3,4-dimethoxystyryl)-2,3-dimethoxystyryl)aniline	$C_{26}H_{27}NO_3$	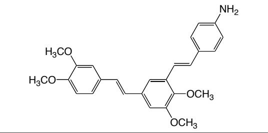	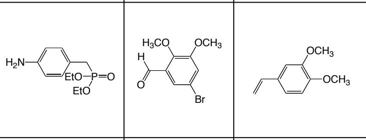		
WH088_F13	4-(5-(3,4-dimethoxystyryl)-2,4-dimethoxystyryl)aniline	$C_{26}H_{27}NO_4$	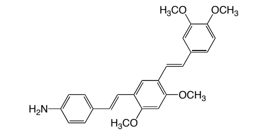	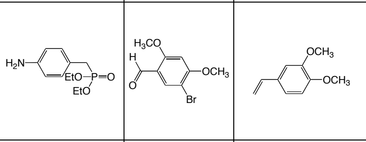		
WH089_F14	4-(3-(3,4-dimethoxystyryl)-4,5-dimethoxystyryl)aniline	$C_{26}H_{27}NO_4$	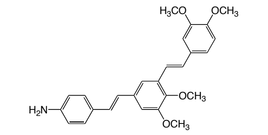	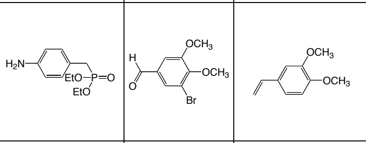		
WH090_F15	4-(2-(3,4-dimethoxystyryl)-4,5-dimethoxystyryl)aniline	$C_{26}H_{27}NO_4$	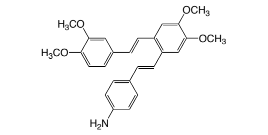	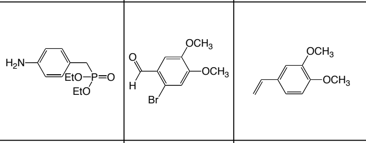		
WH107_H2	4-(3-fluoro-4-(3-methylstyryl)styryl)aniline	$C_{23}H_{20}FN$	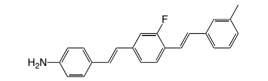	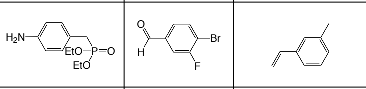		
WH110_H5	4-((E)-2-(5-(3-methylstyryl)benzo[d][1,3]dioxol-4-yl)vinyl)aniline	$C_{24}H_{21}NO_2$	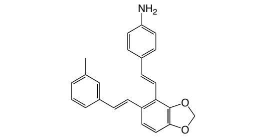	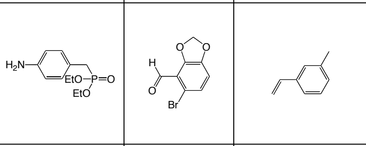		
WH111_H6	4-(4-methyl-2-(3-methylstyryl)styryl)aniline	$C_{24}H_{23}NO$	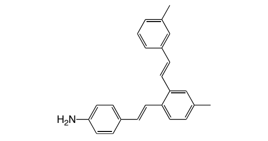	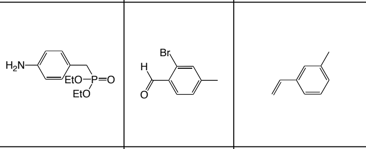		
WH112_H7	4-(5-methoxy-2-(3-methylstyryl)styryl)aniline	$C_{24}H_{23}N$	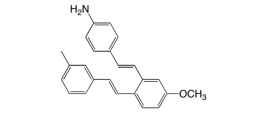	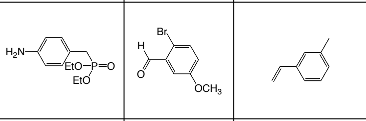		
WH113_H8	4-(4-methoxy-3-(3-methylstyryl)styryl)aniline	$C_{24}H_{23}NO$	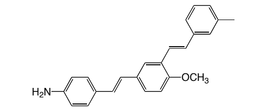	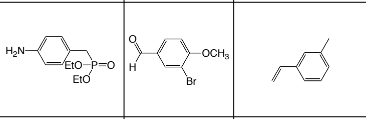		
WH114_H9	4-(2-methoxy-4-(3-methylstyryl)styryl)aniline	$C_{24}H_{23}NO$	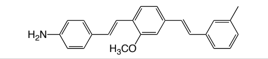	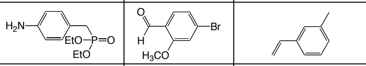		
WH115_H10	4-(2-methoxy-5-(3-methylstyryl)styryl)aniline	$C_{24}H_{23}NO$	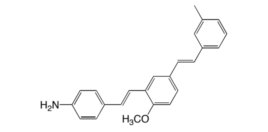	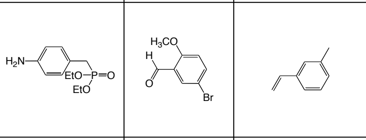		
WH117_H12	4-(2,3-dimethoxy-5-(3-methylstyryl)styryl)aniline	$C_{25}H_{25}NO_2$	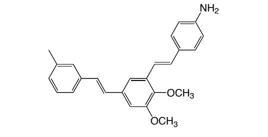	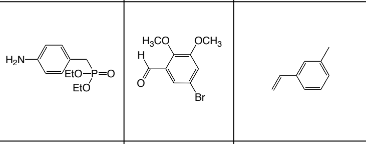		
WH118_H13	4-(2,4-dimethoxy-5-(3-methylstyryl)styryl)aniline	$C_{25}H_{25}NO_2$	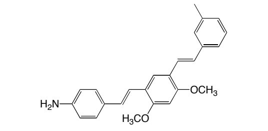	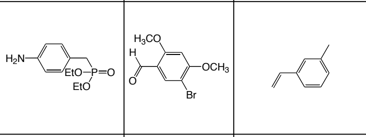		
WH119_H14	4-(3,4-dimethoxy-5-(3-methylstyryl)styryl)aniline	$C_{25}H_{25}NO_2$	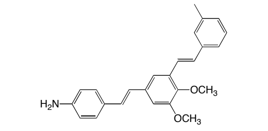	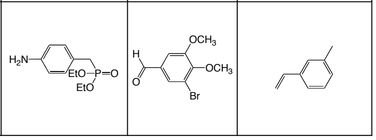		

WH120_H15	4-(4,5-dimethoxy-2-(3-methylstyryl)styryl)aniline	$C_{25}H_{25}NO_2$	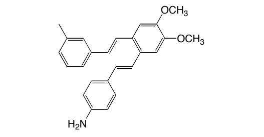	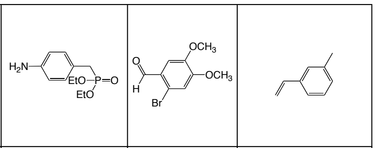		
WH137_J2	3-(4-(4-aminostyryl)-2-fluorostyryl)aniline	$C_{22}H_{19}FN_2$	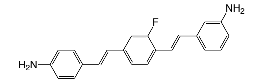	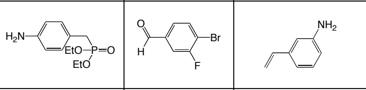		
WH140_J5	3-((E)-2-(4-(4-aminostyryl)benzo[d][1,3]dioxol-5-yl)vinyl)aniline	$C_{23}H_{20}N_2O_2$	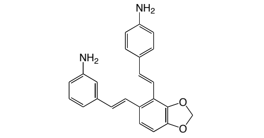	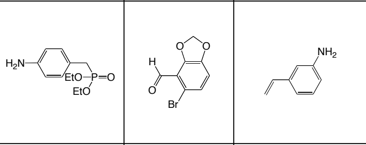		
WH141_J6	3-(2-(4-aminostyryl)-5-methylstyryl)aniline	$C_{23}H_{22}N_2$	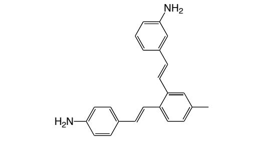	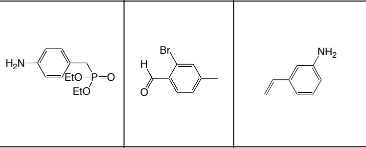		
WH142_J7	3-(2-(4-aminostyryl)-4-methoxystyryl)aniline	$C_{23}H_{22}N_2O$	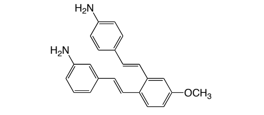	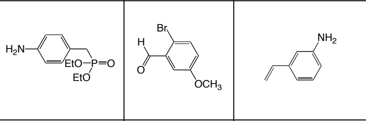		
WH143_J8	3-(5-(4-aminostyryl)-2-methoxystyryl)aniline	$C_{23}H_{22}N_2O$	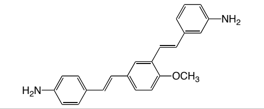	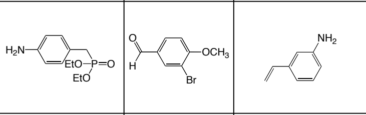		
WH144_J9	3-(4-(4-aminostyryl)-3-methoxystyryl)aniline	$C_{23}H_{22}N_2O$	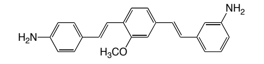	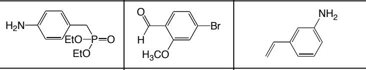		
WH145_J10	3-(3-(4-aminostyryl)-4-methoxystyryl)aniline	$C_{23}H_{22}N_2O$	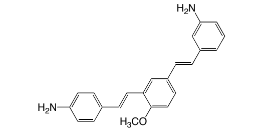	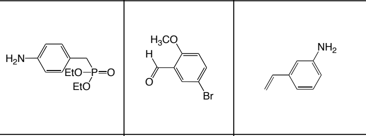		
WH147_J12	3-(3-(4-aminostyryl)-4,5-dimethoxystyryl)aniline	$C_{24}H_{24}N_2O_2$	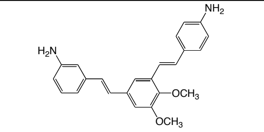	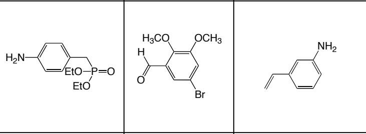		
WH148_J13	3-(5-(4-aminostyryl)-2,4-dimethoxystyryl)aniline	$C_{24}H_{24}N_2O_2$	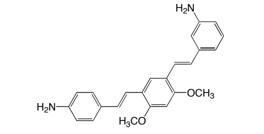	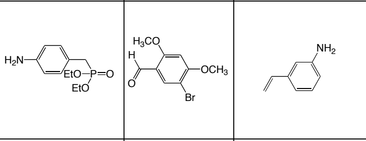		
WH149_J14	3-(5-(4-aminostyryl)-2,3-dimethoxystyryl)aniline	$C_{24}H_{24}N_2O_2$	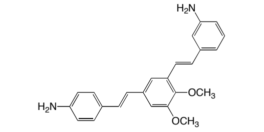	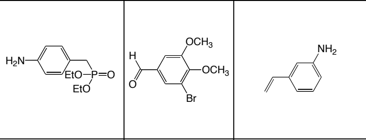		
WH150_J15	3-(2-(4-aminostyryl)-4,5-dimethoxystyryl)aniline	$C_{24}H_{24}N_2O_2$	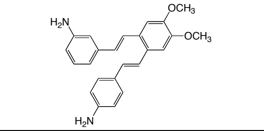	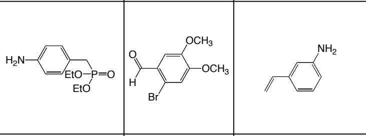		
WH152_K2	4-(3-fluoro-4-(4-methoxystyryl)styryl)aniline	$C_{23}H_{20}FNO$	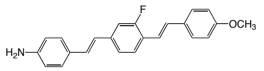	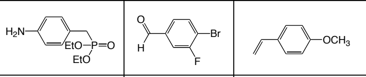		
WH155_K5	4-((E)-2-(5-(4-methoxystyryl)benzo[d][1,3]dioxol-4-yl)vinyl)aniline	$C_{24}H_{21}NO_3$	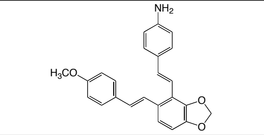	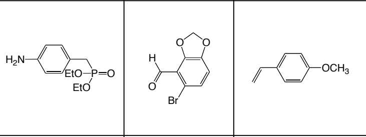		
WH156_K6	4-(2-(4-methoxystyryl)-4-methylstyryl)aniline	$C_{24}H_{23}N$	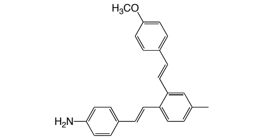	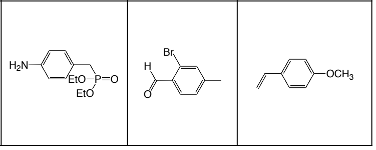		

WH157_K7	4-(5-methoxy-2-(4-methoxystyryl)styryl)aniline	$C_{24}H_{23}NO_2$	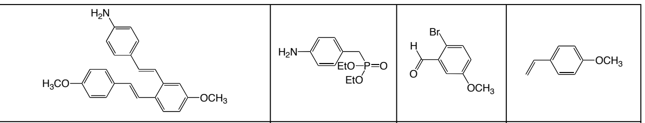			
WH158_K8	4-(4-methoxy-3-(4-methoxystyryl)styryl)aniline	$C_{24}H_{23}NO_2$	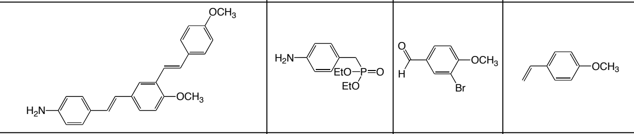			
WH159_K9	4-(2-methoxy-4-(4-methoxystyryl)styryl)aniline	$C_{24}H_{23}NO_2$	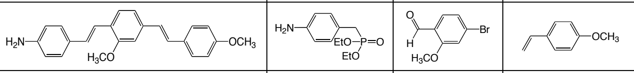			
WH160_K10	4-(2-methoxy-5-(4-methoxystyryl)styryl)aniline	$C_{24}H_{23}NO_2$	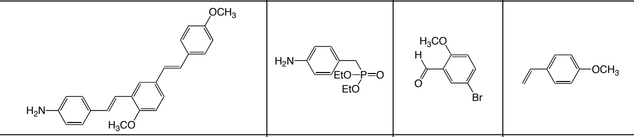			
WH162_K12	4-(2,3-dimethoxy-5-(4-methoxystyryl)styryl)aniline	$C_{25}H_{25}NO_3$	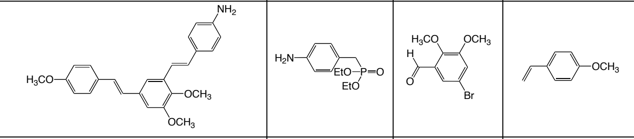			
WH163_K13	4-(2,4-dimethoxy-5-(4-methoxystyryl)styryl)aniline	$C_{25}H_{25}NO_3$	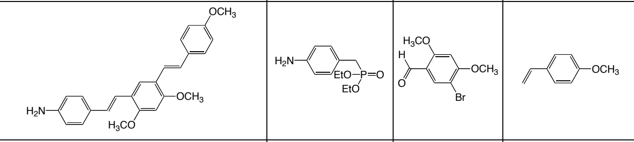			
WH164_K14	4-(3,4-dimethoxy-5-(4-methoxystyryl)styryl)aniline	$C_{25}H_{25}NO_3$	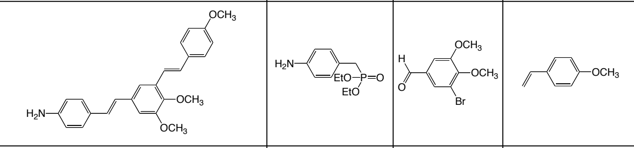			
WH165_K15	4-(4,5-dimethoxy-2-(4-methoxystyryl)styryl)aniline	$C_{25}H_{25}NO_3$	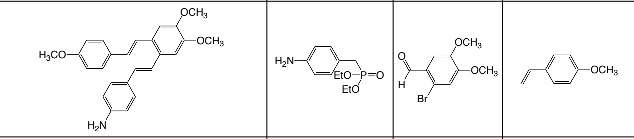			
WH167_L2	4-(4-(4-aminostyryl)-2-fluorostyryl)phenol	$C_{22}H_{18}FNO$	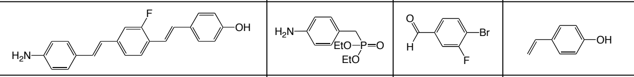			
WH170_L5	4-((E)-2-(4-(4-aminostyryl)benzo[d][1,3]dioxol-5-yl)vinyl)phenol	$C_{23}H_{19}NO_3$	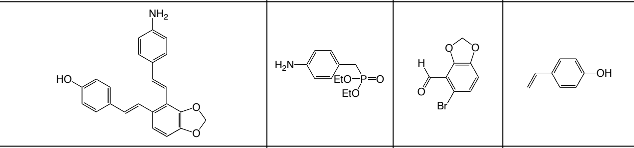			
WH171_L6	(E)-4-(2-(4-aminostyryl)-5-methylphenethyl)phenol	$C_{23}H_{23}NO$	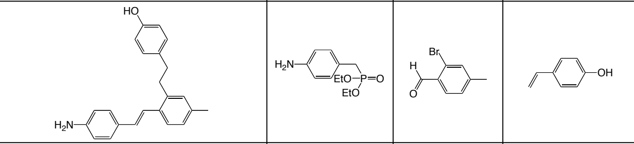			
WH172_L7	4-(2-(4-aminostyryl)-4-methoxystyryl)phenol	$C_{23}H_{21}NO_2$	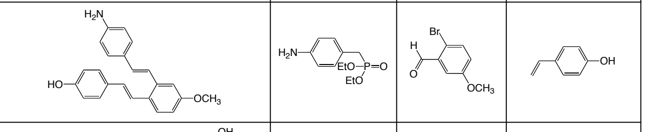			
WH173_L8	4-(5-(4-aminostyryl)-2-methoxystyryl)phenol	$C_{23}H_{21}NO_2$	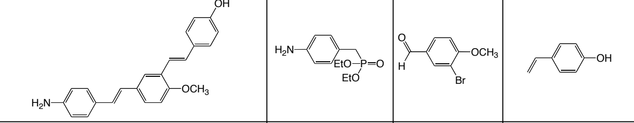			
WH174_L9	4-(4-(4-aminostyryl)-3-methoxystyryl)phenol	$C_{23}H_{21}NO_2$	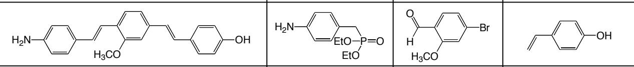			
WH175_L10	4-(3-(4-aminostyryl)-4-methoxystyryl)phenol	$C_{23}H_{21}NO_2$	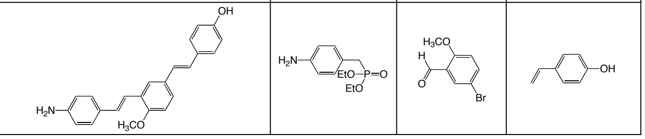			

WH177_L12	4-(3-(4-aminostyryl)-4,5-dimethoxystyryl)phenol	$C_{24}H_{23}NO_3$	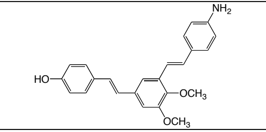	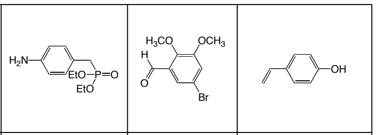
WH178_L13	4-(5-(4-aminostyryl)-2,4-dimethoxystyryl)phenol	$C_{24}H_{23}NO_3$	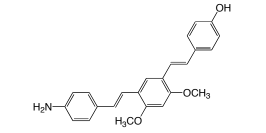	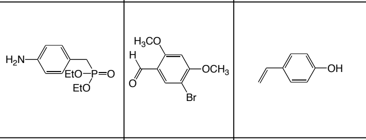
WH179_L14	4-(5-(4-aminostyryl)-2,3-dimethoxystyryl)phenol	$C_{24}H_{23}NO_3$	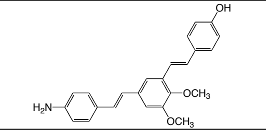	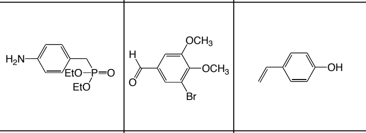
WH180_L15	4-(2-(4-aminostyryl)-4,5-dimethoxystyryl)phenol	$C_{24}H_{23}NO_3$	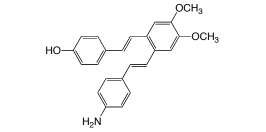	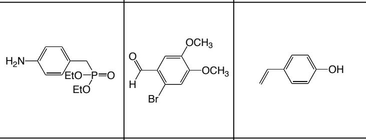
WH182_M2	4-(4-((E)-2-(biphenyl-4-yl)vinyl)-3-fluorostyryl)aniline	$C_{28}H_{22}FN$	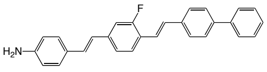	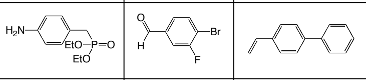
WH185_M5	4-((E)-2-(5-((E)-2-(biphenyl-4-yl)vinyl)benzo[d][1,3]dioxol-4-yl)vinyl)aniline	$C_{29}H_{23}NO_2$	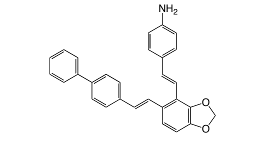	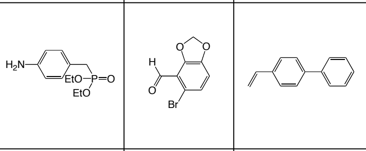
WH186_M6	4-(2-((E)-2-(biphenyl-4-yl)vinyl)-4-methylstyryl)aniline	$C_{29}H_{25}N$	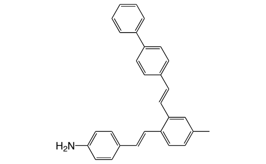	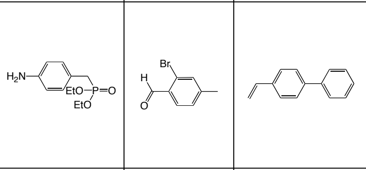
WH187_M7	4-(2-((E)-2-(biphenyl-4-yl)vinyl)-5-methoxystyryl)aniline	$C_{29}H_{25}NO$	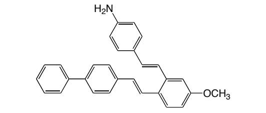	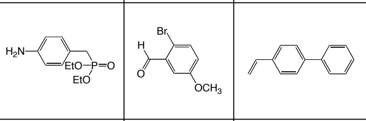
WH188_M8	4-(3-((E)-2-(biphenyl-3-yl)vinyl)-4-methoxystyryl)aniline	$C_{29}H_{25}NO$	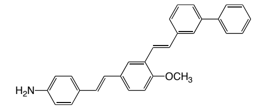	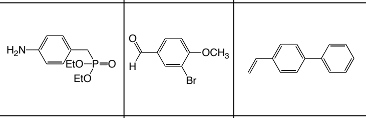
WH189_M9	4-(4-((E)-2-(biphenyl-4-yl)vinyl)-2-methoxystyryl)aniline	$C_{29}H_{25}NO$	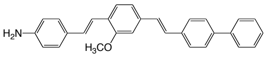	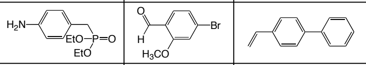
WH190_M10	4-(5-((E)-2-(biphenyl-4-yl)vinyl)-2-methoxystyryl)aniline	$C_{29}H_{25}NO$	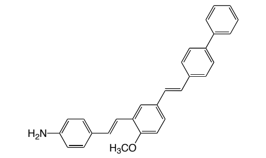	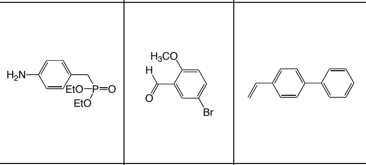
WH192_M12	4-(5-((E)-2-(biphenyl-4-yl)vinyl)-2,3-dimethoxystyryl)aniline	$C_{30}H_{27}NO_2$	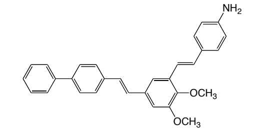	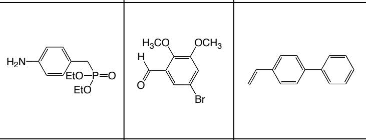
WH193_M13	4-(5-((E)-2-(biphenyl-4-yl)vinyl)-2,4-dimethoxystyryl)aniline	$C_{30}H_{27}NO_2$	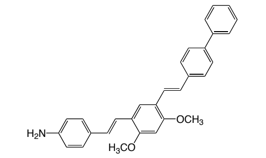	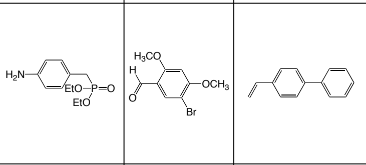
WH194_M14	4-(3-((E)-2-(biphenyl-4-yl)vinyl)-4,5-dimethoxystyryl)aniline	$C_{30}H_{27}NO_2$	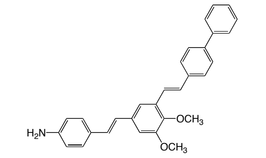	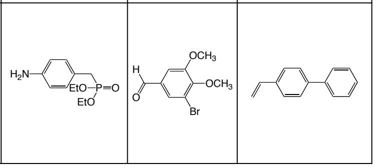

WH195_M15	4-(2-((E)-2-(biphenyl-4-yl)vinyl)-4,5-dimethoxystyryl)aniline	$C_{30}H_{27}NO_2$	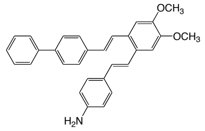	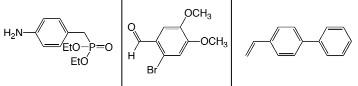
WH197_N2	4-(4-((E)-2-(anthracen-9-yl)vinyl)-3-fluorostyryl)aniline	$C_{30}H_{22}FN$	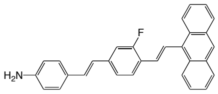	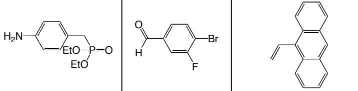
WH200_N5	4-((E)-2-(5-((E)-2-(1,2-dihydroanthracen-9-yl)vinyl)benzo[d][1,3]dioxol-4-yl)vinyl)anili	$C_{31}H_{25}NO_2$	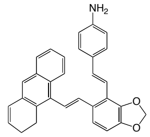	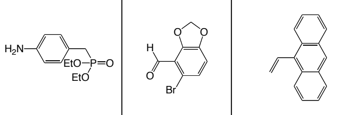
WH201_N6	4-(2-((E)-2-(anthracen-9-yl)vinyl)-4-methylstyryl)aniline	$C_{31}H_{25}N$	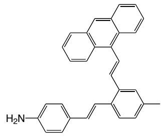	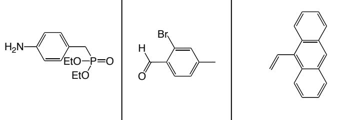
WH202_N7	4-(2-((E)-2-(anthracen-9-yl)vinyl)-5-methoxystyryl)aniline	$C_{31}H_{25}NO$	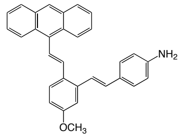	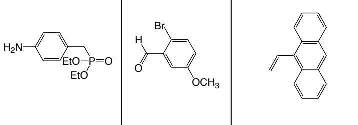
WH203_N8	4-(3-((E)-2-(anthracen-9-yl)vinyl)-4-methoxystyryl)aniline	$C_{31}H_{25}NO$	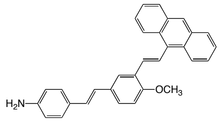	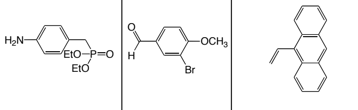
WH204_N9	4-(4-((E)-2-(anthracen-9-yl)vinyl)-2-methoxystyryl)aniline	$C_{31}H_{25}NO$	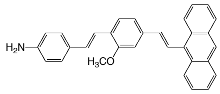	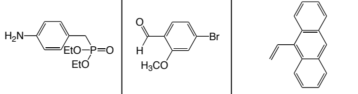
WH205_N10	4-(5-((E)-2-(anthracen-9-yl)vinyl)-2-methoxystyryl)aniline	$C_{31}H_{25}NO$	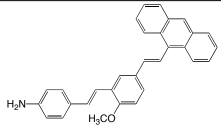	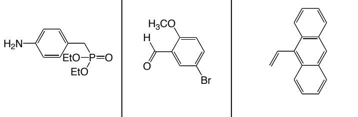
WH207_N12	4-(5-((E)-2-(anthracen-9-yl)vinyl)-2,3-dimethoxystyryl)aniline	$C_{32}H_{27}NO_2$	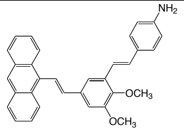	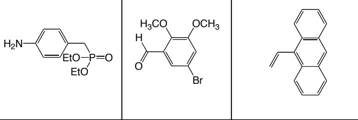
WH208_N13	4-(5-((E)-2-(anthracen-9-yl)vinyl)-2,4-dimethoxystyryl)aniline	$C_{32}H_{27}NO_2$	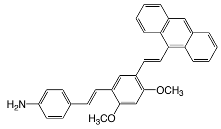	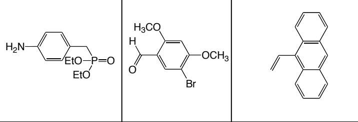
WH209_N14	4-(3-((E)-2-(anthracen-9-yl)vinyl)-4,5-dimethoxystyryl)aniline	$C_{32}H_{27}NO_2$	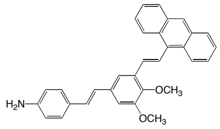	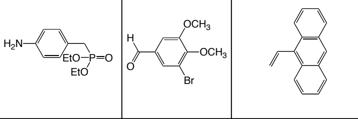
WH210_N15	4-(2-((E)-2-(anthracen-9-yl)vinyl)-4,5-dimethoxystyryl)aniline	$C_{32}H_{27}NO_2$	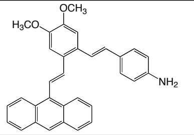	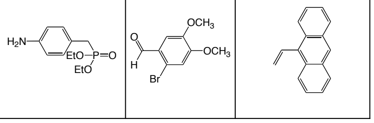
