# Supplementary material for: Structure-Activity Relationship of Nerve-Highlighting Fluorophores
Source: PLoS One. 2013 Sep 9;8(9):e73493. doi: 10.1371/journal.pone.0073493 (PMC3767781; doi:10.1371/journal.pone.0073493)
Supplement: Table S2 — (PDF) [file pone.0073493.s004.pdf]

Table S2 - Chemical Building Blocks

Chemical Name	CAS#	MW	Chemical Formula	Manufacturer	Portion of Molecule	Building Block Type	Structure
diethyl 4-aminobenzylphosphonate	20074-79-7	243.24	$C_{11}H_{18}NO_3P$	Acros Organics	Left	Diethyl Phosphonate	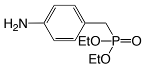
4-aminostyrene	1520-21-4	119.16	C_8H_9N	Sigma Aldrich	Left	Styrene	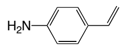
2-bromo-4-methylbenzaldehyde	824-54-4	199.04	C_8H_7BrO	Sigma Aldrich	Middle	Bromo Benzaldhyde	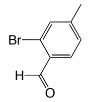
2-bromo-5-methoxybenzaldehyde	7507-86-0	215.04	$C_8H_7BrO_2$	Sigma Aldrich	Middle	Bromo Benzaldhyde	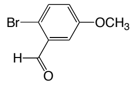
3-bromo-4-methyoxybenzaldehyde	34841-06-0	215.04	$C_8H_7BrO_2$	Sigma Aldrich	Middle	Bromo Benzaldhyde	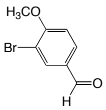
4-bromo-2-methoxybenzaldehyde	43192-33-2	215.04	$C_8H_7BrO_2$	Sigma Aldrich	Middle	Bromo Benzaldhyde	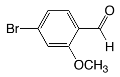
5-bromo-2-methoxybenzaldehyde	25016-01-7	215.04	$C_8H_7BrO_2$	Sigma Aldrich	Middle	Bromo Benzaldhyde	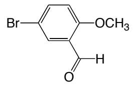
5-bromo-2-ethoxybenzaldehyde	79636-94-5	229.98	$C_9H_9BrO_2$	Maybridge	Middle	Bromo Benzaldhyde	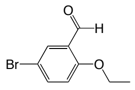
5-bromo-2,3-dimethoxybenzaldehyde	71295-21-1	245.07	$C_9H_9BrO_3$	Sigma Aldrich	Middle	Bromo Benzaldhyde	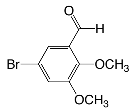
5-bromo-2,4-dimethoxybenzaldehyde	130333-46-9	245.07	$C_9H_9BrO_3$	Sigma Aldrich	Middle	Bromo Benzaldhyde	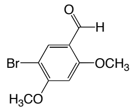
3-bromo-4,5-dimethoxybenzaldehyde	6948-30-7	245.07	$C_9H_9BrO_3$	Sigma Aldrich	Middle	Bromo Benzaldhyde	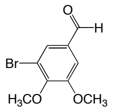
2-bromo-4,5-dimethoxybenzaldehyde	5392-10-9	245.07	$C_9H_9BrO_3$	Sigma Aldrich	Middle	Bromo Benzaldhyde	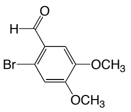
4-bromo-3-fluorobenzaldehyde	133059-43-5	203.01	C_7H_4BrFO	Sigma Aldrich	Middle	Bromo Benzaldhyde	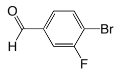
5-bromo-1,3-benzodioxole-4-carboxaldehyde	72744-54-8	229.03	$C_8H_5BrO_3$	Sigma Aldrich	Middle	Bromo Benzaldhyde	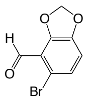
diethy (4-cyanobenzyl)phosphonate	1552-41-6	253.23	$C_{12}H_{16}NO_3P$	TCI America	Right	Diethyl Phosphonate	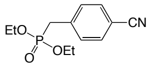
diethyl (4-fluorobenzyl)phosphonate	63909-58-0	246.08	$C_{11}H_{16}FO_3P$	TCI America	Right	Diethyl Phosphonate	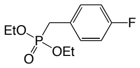

diethyl (4-isopropylbenzyl)phosphonate	77237-55-9	270.30	$C_{14}H_{23}O_3P$	TCI America	Right	Diethyl Phosphonate	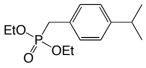
diethyl (4-chlorobenzyl)phosphonate	39225-17-7	262.67	$C_{11}H_{16}ClO_3P$	TCI America	Right	Diethyl Phosphonate	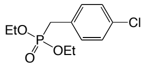
diethyl (4-bromobenzyl)phosphonate	38186-51-5	307.12	$C_{11}H_{16}BrO_3P$	TCI America	Right	Diethyl Phosphonate	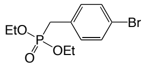
diethyl (4-iodobenzyl)phosphonate	173443-43-1	354.12	$C_{11}H_{16}IO_3P$	TCI America	Right	Diethyl Phosphonate	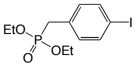
diethyl (4-methoxybenzyl)phosphonate	1145-93-3	258.25	$C_{12}H_{19}O_4P$	TCI America	Right	Diethyl Phosphonate	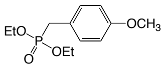
diethyl (4-methylbenzyl)phosphonate	3762-25-2	242.25	$C_{12}H_{19}O_3P$	Sigma Aldrich	Right	Diethyl Phosphonate	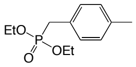
diethyl (2-methylbenzyl)phosphonate	62778-16-9	242.25	$C_{12}H_{19}O_3P$	TCI America	Right	Diethyl Phosphonate	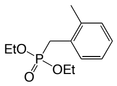
diethyl (3-methylbenzyl)phosphonate	63909-50-2	242.25	$C_{12}H_{19}O_3P$	TCI America	Right	Diethyl Phosphonate	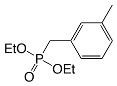
diethyl (4-aminobenzyl)phosphonate	20074-79-7	243.24	$C_{11}H_{18}NO_3P$	Acros Organics	Right	Diethyl Phosphonate	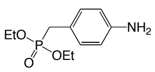
diethyl (3-chlorobenzyl)phosphonate	78055-64-8	262.67	$C_{11}H_{16}ClO_3P$	TCI America	Right	Diethyl Phosphonate	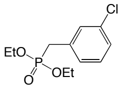
2,4-dimethylstyrene	2234-20-0	132.2	$C_{10}H_{12}$	Acros Organics	Right	Styrene	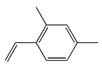
4-aminostyrene (Boc protected)	1520-21-4	119.16	C_8H_9N	Sigma Aldrich	Right	Styrene	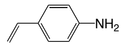
2-vinylnaphthalene	827-54-3	154.08	$C_{12}H_{10}$	Sigma Aldrich	Right	Styrene	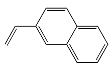
3,4-dimethoxystyrene	6380-23-0	164.08	$C_{10}H_{12}O_2$	Sigma Aldrich	Right	Styrene	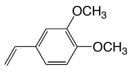
3-methylstyrene	100-80-1	118.18	C_9H_{10}	Sigma Aldrich	Right	Styrene	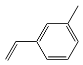
3-aminostryene (BOC protected)	15411-43-5	119.16	C_8H_9N	Sigma Aldrich	Right	Styrene	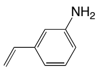
4-methoxystyrene	637-69-4	134.18	$C_9H_{10}O$	Sigma Aldrich	Right	Styrene	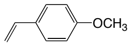
4-tert-butylstyrene	1746-23-2	176.12	$C_{12}H_{16}O$	Sigma Aldrich	Right	Styrene	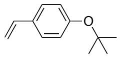
4-vinylbiphenyl	2350-89-2	180.25	$C_{14}H_{12}$	Sigma Aldrich	Right	Styrene	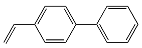
9-vinylanthracene	2444-68-0	204.27	$C_{16}H_{12}$	Sigma Aldrich	Right	Styrene	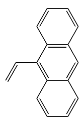
