# Supplementary material for: Structure-Activity Relationship of Nerve-Highlighting Fluorophores
Source: PLoS One. 2013 Sep 9;8(9):e73493. doi: 10.1371/journal.pone.0073493 (PMC3767781; doi:10.1371/journal.pone.0073493)
Supplement: Table S3 — (PDF) [file pone.0073493.s005.pdf]

Table S3 – Physiochemical Properties

**Fluorophore Name**	*Molecular Weight*	*LogD (pH 7.4)*	*Retention Time (min)*	*Yield (mg)*	*Purity (%)*
HW006_A6	336.4	5.61	21.9	10.5	13.7
HW007_A7	352.4	5.52	19.5	16.8	54.7
HW008_A8	352.4	5.52	19.7	11.8	28.4
HW009_A9	352.4	5.52	20.7	10.1	69.8
HW010_A10	352.4	5.52	18.6	16.2	1.4
HW011_A11	366.5	5.88	20.3	14.7	3.2
HW012_A12	383.1	5.36	18.3	14.0	1.7
HW013_A13	383.1	5.36	19.1	16.0	27.5
HW014_A14	383.1	5.36	19.1	13.6	32.8
HW015_A15	383.1	5.36	17.6	10.1	61.2
HW021_B6	329.4	6.48	24.0	16.8	64.1
HW022_B7	345.4	5.81	21.6	23.5	42.4
HW023_B8	345.4	5.81	22.4	17.0	22.3
HW024_B9	345.4	5.81	23.2	15.2	28.2
HW025_B10	345.4	5.81	22.3	20.6	31.6
HW026_B11	359.4	6.17	23.8	27.6	34.4
HW027_B12	375.4	5.65	21.6	25.7	32.4
HW028_B13	375.4	5.65	23.1	21.8	1.5
HW029_B14	375.4	5.65	21.7	22.5	44.6
HW030_B15	375.4	5.65	19.5	28.2	36.3
HW036_C6	353.2	7.58	26.3	14.7	33.9
HW037_C7	369.5	6.91	24.2	19.2	12.9
HW038_C8	369.5	6.91	25.1	16.9	16.4
HW039_C9	369.5	6.91	25.7	13.4	27.0
HW040_C10	369.5	6.91	25.0	24.8	32.3
HW041_C11	383.5	7.27	26.2	26.8	43.9
HW042_C12	399.5	6.75	24.4	25.5	38.8
HW043_C13	399.5	6.57	26.7	17.3	2.5
HW044_C14	399.5	6.57	24.4	17.6	37.0
HW045_C15	399.5	6.75	19.6	24.5	5.5
HW051_D6	345.9	6.94	25.0	17.0	65.0
HW052_D7	361.9	6.27	22.5	20.9	31.3
HW053_D8	361.9	6.27	23.8	24.2	23.5
HW054_D9	361.9	6.27	24.7	13.5	48.6
HW025_D10	361.9	6.27	23.6	15.3	19.1
HW056_D11	375.9	6.63	25.2	23.7	44.5
HW057_D12	391.9	6.11	23.1	27.8	40.4
HW058_D13	391.9	6.31	23.7	14.4	1.3
HW059_D14	391.9	6.11	23.0	19.3	46.1
HW060_D15	391.9	6.11	22.7	18.3	7.3
HW066_E6	390.3	7.11	25.4	15.6	34.4

HW067_E7	406.3	6.43	22.9	21.3	3.1
HW068_E8	406.3	6.43	24.2	12.4	8.6
HW069_E9	406.3	6.43	25.2	10.7	41.6
HW070_E10	406.3	6.43	24.2	17.3	18.7
HW071_E11	420.3	6.79	25.6	28.6	58.5
HW072_E12	436.4	6.28	23.5	25.4	23.4
HW073_E13	436.4	6.47	25.3	18.1	1.1
HW074_E14	436.4	6.28	23.4	16.9	33.9
HW075_E15	436.4	6.28	18.5	22.1	5.6
HW081_F6	437.3	7.27	25.8	16.7	30.6
HW082_F7	453.3	6.60	23.4	19.2	9.0
HW083_F8	453.3	6.60	24.7	16.4	7.5
HW084_F9	453.3	6.60	25.6	9.5	11.8
HW085_F10	453.3	6.60	24.7	50.6	34.6
HW086_F11	467.3	6.95	25.9	48.2	31.1
HW087_F12	483.3	6.44	24.0	51.9	30.0
HW088_F13	483.3	6.44	24.0	40.4	3.4
HW089_F14	483.3	6.44	23.9	15.8	42.3
HW090_F15	483.3	6.44	21.7	36.8	54.8
HW096_G6	341.6	6.18	20.2	15.4	47.7
HW097_G7	357.4	5.51	17.4	15.8	21.2
HW098_G8	357.4	5.51	21.2	12.3	18.7
HW099_G9	357.4	5.51	22.1	10.1	32.6
HW100_G10	357.4	5.51	21.3	13.8	12.4
HW101_G11	371.5	5.87	23.5	24.5	10.3
HW102_G12	387.5	5.35	20.1	20.5	22.4
HW103_G13	387.5	5.35	16.6	11.6	12.5
HW104_G14	387.5	5.35	19.9	16.4	32.8
HW105_G15	387.5	5.35	15.3	20.6	18.6
HW111_H6	325.5	6.85	24.3	11.6	9.1
HW112_H7	341.5	6.18	21.7	18.1	15.7
HW113_H8	341.5	6.18	24.8	4.3	9.8
HW114_H9	341.5	6.18	25.3	7.3	18.9
HW115_H10	341.5	6.18	22.5	25.0	28.8
HW116_H11	355.5	6.54	23.9	21.7	11.0
HW117_H12	371.5	6.02	22.1	22.2	31.8
HW118_H13	371.5	6.02	18.6	12.1	0.8
HW119_H14	371.5	6.02	22.2	16.1	52.5
HW120_H15	371.5	6.02	19.7	24.9	23.8
HW126_I6	325.5	6.85	24.1	10.5	11.5
HW127_I7	341.5	6.18	21.6	16.8	23.2
HW128_I8	341.45	6.18	21.8	11.3	1.1
HW129_I9	341.5	6.18	23.3	8.4	41.0
HW130_I10	341.5	6.18	22.6	23.0	49.1
HW131_I11	355.5	6.54	24.0	19.9	53.2

HW132_I12	371.5	6.02	21.8	19.1	24.6
HW133_I13	371.5	6.02	24.6	17.2	0.9
HW134_I14	371.5	6.02	21.9	18.2	55.4
HW135_I15	371.5	6.02	19.7	28.2	47.2
HW141_J6	325.5	6.85	24.4	17.1	63.6
HW142_J7	341.5	6.18	21.8	19.7	26.9
HW143_J8	341.5	6.18	22.7	24.5	10.6
HW144_J9	341.5	6.18	23.6	15.9	25.5
HW145_J10	341.5	6.18	22.4	23.2	17.9
HW146_J11	355.5	6.54	23.9	18.3	31.4
HW147_J12	371.5	6.02	21.9	26.9	35.4
HW148_J13	371.5	6.02	26.4	28.1	3.5
HW149_J14	371.5	6.02	22.1	21.8	36.2
HW150_J15	371.5	6.02	19.6	25.1	30.6
HW156_K6	326.4	5.51	17.7	18.9	74.8
HW157_K7	342.4	4.84	14.9	18.1	35.9
HW158_K8	342.4	4.84	14.1	13.5	36.5
HW159_K9	342.4	4.84	13.9	9.7	69.6
HW160_K10	342.4	4.84	14.3	18.0	90.7
HW161_K11	356.5	5.19	16.3	29.7	86.1
HW162_K12	372.5	4.68	13.5	25.2	30.7
HW163_K13	372.5	4.68	13.2	13.2	72.4
HW164_K14	372.5	4.68	13.5	15.0	40.6
HW165_K15	372.5	4.68	11.8	21.3	34.3
HW171_L6	345.9	6.94	24.7	12.0	9.3
HW172_L7	361.9	6.27	22.1	18.6	5.0
HW173_L8	361.9	6.27	23.3	9.4	4.6
HW174_L9	361.9	6.27	24.3	6.1	11.3
HW175_L10	361.9	6.27	23.2	8.9	1.0
HW176_L11	375.9	6.63	24.8	9.6	47.8
HW177_L12	391.9	6.11	22.7	28.6	33.6
HW178_L13	391.9	6.11	25.2	16.7	2.4
HW179_L14	391.9	6.11	22.5	10.3	5.4
HW180_L15	391.9	6.11	20.2	17.8	26.9
WH017_B2	343.4	6.99	26.1	27.4	39.7
WH020_B5	369.5	6.47	23.1	24.9	43.4
WH021_B6	339.5	7.36	25.9	24.1	2.3
WH022_B7	355.5	6.69	23.9	26.2	50.5
WH023_B8	355.5	6.69	24.0	37.6	48.6
WH024_B9	355.5	6.69	24.6	30.6	52.9
WH025_B10	355.5	6.69	26.4	26.3	51.9
WH027_B12	385.5	6.54	23.6	36.1	69.2
WH028_B13	385.5	6.54	23.4	36.5	6.5
WH029_B14	385.5	6.54	23.3	31.6	16.1
WH030_B15	385.5	6.54	22.8	23.2	43.9

WH047_D2	330.4	5.14	16.4	28.3	60.0
WH050_D5	356.4	4.62	14.4	19.9	1.3
WH051_D6	328.5	5.70	15.3	26.9	19.5
WH052_D7	342.4	4.84	15.3	25.2	79.5
WH053_D8	342.4	4.84	14.0	24.6	78.6
WH054_D9	342.4	4.84	14.7	32.1	84.6
WH055_D10	342.4	4.84	14.2	26.6	73.1
WH057_D12	372.5	4.68	14.1	32.1	86.0
WH058_D13	372.5	4.68	10.6	21.8	84.0
WH059_D14	372.5	4.68	13.4	37.3	24.6
WH060_D15	372.5	4.68	12.1	30.4	65.5
WH062_E2	365.4	6.96	26.8	37.8	25.5
WH065_E5	391.5	6.44	11.9	20.5	0.4
WH066_E6	361.5	7.33	15.2	26.7	5.0
WH067_E7	377.5	6.66	24.4	29.4	42.0
WH068_E8	377.5	6.66	24.3	30.5	45.4
WH069_E9	377.5	6.66	24.8	20.8	70.5
WH070_E10	377.5	6.66	25.4	36.4	51.0
WH072_E12	407.5	6.50	23.6	40.3	77.8
WH073_E13	407.5	6.50	23.4	25.0	3.9
WH074_E14	407.5	6.50	23.3	31.5	2.4
WH075_E15	407.5	6.50	21.3	26.7	85.5
WH077_F2	375.4	5.65	20.2	28.5	30.6
WH080_F5	401.5	5.13	19.6	19.9	1.8
WH081_F6	371.5	6.02	19.7	25.1	7.1
WH082_F7	387.5	5.35	19.2	25.2	60.4
WH083_F8	387.5	5.35	18.0	29.3	36.2
WH084_F9	387.5	5.35	18.7	27.0	66.3
WH085_F10	387.5	5.35	18.8	34.5	71.8
WH087_F12	417.5	5.19	18.7	31.9	72.0
WH088_F13	417.5	5.19	19.5	28.1	22.4
WH089_F14	417.5	5.19	17.9	27.2	69.8
WH090_F15	417.5	5.19	16.7	30.4	42.7
WH107_H2	329.4	6.48	25.5	31.0	36.0
WH110_H5	355.4	5.96	22.6	20.5	1.2
WH111_H6	325.5	6.85	25.4	25.4	3.8
WH112_H7	341.5	6.18	23.4	26.2	62.1
WH113_H8	341.5	6.18	23.5	37.0	45.9
WH114_H9	341.5	6.18	24.1	30.4	64.9
WH115_H10	341.5	6.18	26.0	39.1	45.5
WH117_H12	371.5	6.02	22.9	39.5	57.2
WH118_H13	371.5	6.02	23.3	24.0	1.1
WH119_H14	371.5	6.02	22.7	27.4	21.8
WH120_H15	371.5	6.02	20.5	32.2	59.6
WH137_J2	330.4	5.14	15.0	40.2	32.7

WH140_J5	356.4	4.62	14.5	23.5	2.7
WH141_J6	326.4	5.51	23.1	31.0	9.8
WH142_J7	342.4	4.84	14.7	27.2	87.4
WH143_J8	342.4	4.84	13.1	26.4	67.7
WH144_J9	342.4	4.84	13.3	33.0	66.5
WH145_J10	342.4	4.84	13.1	20.3	59.5
WH147_J12	372.5	4.68	13.0	50.4	56.3
WH148_J13	372.5	4.68	9.5	37.4	21.6
WH149_J14	372.5	4.68	12.8	55.6	15.2
WH150_J15	372.5	4.68	11.7	15.7	69.8
WH152_K2	345.4	5.81	23.0	27.6	36.0
WH155_K5	371.4	5.29	18.4	23.2	1.2
WH156_K6	357.4	6.18	15.0	20.9	29.5
WH157_K7	357.4	5.51	20.9	21.5	29.0
WH158_K8	357.4	5.51	20.3	34.4	12.3
WH159_K9	357.4	5.51	21.1	21.3	53.1
WH160_K10	357.4	5.51	21.3	27.1	41.7
WH162_K12	387.5	5.35	20.1	22.2	51.3
WH163_K13	387.5	5.35	19.6	21.5	8.0
WH164_K14	387.5	5.35	11.6	22.5	8.4
WH165_K15	387.5	5.35	15.8	19.3	30.6
WH167_L2	387.5	5.66	21.5	21.9	4.2
WH170_L5	413.5	5.14	19.6	22.4	2.4
WH171_L6	385.5	6.23	19.4	19.7	5.0
WH172_L7	399.5	5.36	19.6	18.5	8.2
WH173_L8	399.5	5.36	19.6	36.1	7.9
WH174_L9	399.5	5.36	22.4	8.0	1.8
WH175_L10	388.5	5.36	19.5	28.6	11.9
WH177_L12	429.6	5.20	19.6	17.6	6.5
WH178_L13	429.6	5.20	19.5	21.0	10.3
WH179_L14	429.6	5.20	16.7	28.5	11.0
WH180_L15	429.6	5.20	16.7	12.6	7.9
WH182_M2	391.5	7.61	26.9	30.5	68.7
WH185_M5	417.5	7.09	23.1	21.3	7.1
WH186_M6	387.5	7.98	26.8	25.9	6.6
WH187_M7	403.5	7.31	24.8	30.9	46.1
WH188_M8	403.5	7.31	24.9	18.9	42.3
WH189_M9	403.5	7.31	25.5	29.4	67.1
WH190_M10	403.5	7.31	25.1	20.2	39.9
WH192_M12	433.5	7.16	24.4	28.9	70.2
WH193_M13	433.5	7.16	24.2	24.7	4.0
WH194_M14	433.5	7.16	24.2	19.2	7.9
WH195_M15	433.5	7.16	22.4	2.9	49.1
WH197_N2	415.5	7.95	27.0	28.1	4.2
WH200_N5	441.5	7.61	23.1	22.6	4.5

WH201_N6	411.5	8.32	15.0	20.7	31.3
WH202_N7	427.5	7.65	21.1	21.0	13.6
WH203_N8	427.5	7.65	25.9	26.0	52.8
WH204_N9	427.5	7.65	25.9	23.2	11.8
WH205_N10	427.5	7.65	25.8	21.7	1.5
WH207_N12	457.6	7.49	25.6	24.3	41.7
WH208_N13	457.6	7.49	25.2	20.2	35.1
WH209_N14	457.6	7.49	25.2	26.9	2.1
WH210_N15	457.6	7.49	25.1	21.2	1.2
