# Supplementary material for: Structure-Activity Relationship of Nerve-Highlighting Fluorophores
Source: PLoS One. 2013 Sep 9;8(9):e73493. doi: 10.1371/journal.pone.0073493 (PMC3767781; doi:10.1371/journal.pone.0073493)
Supplement: Table S4 — (PDF) [file pone.0073493.s006.pdf]

Table S4 – Spectroscopic Properties

Fluorophore Name	Abs. Maximum (nm)	Fluorescence Maximum (nm)							
		Crude					Purified		
		350 nm	*375 nm*	*400 nm*	*425 nm*	*450 nm*	*MeOH*	*DMSO*	*FBS*
HW006_A6	309	512	512	545	541	X	487	505	446
HW007_A7	319	465	470	470	X	X	514	525	441
HW008_A8	314	455	445	445	X	X	X	470	454
HW009_A9	384	604	604	606	616	613	598	602	544
HW010_A10	314	509	512	519	X	X	487	510	465
HW011_A11	320	493	490	490	X	X	478	504	455
HW012_A12	312	516	511	519	X	X	X	464	457
HW013_A13	356	476	482	X	X	X	499	517	467
HW014_A14	328	467	460	460	X	X	463	469	449
HW015_A15	380	509	512	515	X	X	499	512	455
HW021_B6	339	538	527	545	X	X	X	X	X
HW022_B7	313	554	528	545	X	X	X	X	X
HW023_B8	305	458	458	458	X	X	X	X	X
HW024_B9	379	540	544	544	546	560	506	535	474
HW025_B10	303	540	515	520	X	X	X	X	X
HW026_B11	303	544	527	545	543	538	427	448	435
HW027_B12	319	547	512	526	X	X	X	X	X
HW028_B13	306	477	480	483	487	X	X	X	X
HW029_B14	281	541	526	541	X	X	X	X	X
HW030_B15	294	530	530	543	538	531	X	X	X
HW036_C6	303	528	528	525	527	X	X	X	X
HW037_C7	300	545	541	545	545	X	X	X	X
HW038_C8	344	465	446	446	X	X	426	433	434
HW039_C9	378	532	533	539	540	562	438	449	449
HW040_C10	320	537	527	519	525	X	424	437	439
HW041_C11	319	536	527	527	X	X	423	439	442
HW042_C12	318	539	527	520	X	X	X	X	X
HW043_C13	343	460	459	462	465	X	X	X	X
HW044_C14	290	511	511	515	X	X	X	X	X
HW045_C15	330	546	550	556	575	580	X	X	X
HW051_D6	316	558	555	552	554	X	X	X	X
HW052_D7	295	565	553	567	570	567	X	X	X
HW053_D8	294	450	483	544	560	565	X	X	X
HW054_D9	377	560	562	567	567	574	436	452	452
HW025_D10	302	444	440	443	X	X	X	X	X
HW056_D11	300	448	440	441	X	X	442	458	453
HW057_D12	289	447	439	441	X	X	X	X	X
HW058_D13	304	471	470	468	488	483	X	X	X
HW059_D14	296	451	439	442	X	X	X	X	X

HW060_D15	283	559	561	570	576	576	X	X	X
HW066_E6	291	551	551	545	554	X	X	X	X
HW067_E7	330	570	566	567	577	569	X	X	X
HW068_E8	322	451	441	447	X	X	X	X	X
HW069_E9	382	564	564	566	568	573	439	453	455
HW070_E10	327	445	447	447	562	550	X	X	X
HW071_E11	302	456	439	442	X	X	444	460	449
HW072_E12	307	453	440	446	X	X	X	X	X
HW073_E13	320	470	483	472	X	X	X	X	X
HW074_E14	317	457	448	449	534	X	X	X	X
HW075_E15	287	569	570	573	578	583	522	544	438
HW081_F6	307	467	484	519	543	X	X	X	X
HW082_F7	312	455	445	459	570	X	X	X	X
HW083_F8	322	456	440	446	X	X	X	X	X
HW084_F9	383	570	574	574	577	579	442	458	457
HW085_F10	319	447	448	448	X	X	X	X	X
HW086_F11	320	460	449	456	X	X	X	X	X
HW087_F12	307	457	444	448	X	X	X	X	X
HW088_F13	340	464	461	458	X	X	X	X	X
HW089_F14	312	456	441	441	448	X	X	X	X
HW090_F15	347	520	527	544	552	X	X	X	X
HW096_G6	330	506	508	508	X	X	X	X	X
HW097_G7	324	540	542	544	563	567	X	X	X
HW098_G8	344	458	455	454	X	X	X	450	436
HW099_G9	340	520	520	523	525	558	450	456	454
HW100_G10	323	496	481	484	X	X	X	X	X
HW101_G11	331	520	511	518	525	X	423	428	4533
HW102_G12	328	512	511	519	X	X	X	X	X
HW103_G13	261	452	454	458	X	X	X	X	X
HW104_G14	317	470	457	455	X	X	X	X	X
HW105_G15	356	513	515	522	563	572	X	X	X
HW111_H6	296	534	535	536	542	X	449	513	440
HW112_H7	329	553	553	553	556	X	X	X	X
HW113_H8	348	466	457	458	X	X	431	442	434
HW114_H9	342	537	538	541	542	570	439	449	452
HW115_H10	332	531	527	527	532	X	425	440	452
HW116_H11	315	534	527	534	532	X	425	440	446
HW117_H12	319	539	526	544	X	X	X	X	X
HW118_H13	326	473	483	483	492	X	X	X	X
HW119_H14	322	509	511	507	X	X	X	X	X
HW120_H15	380	525	528	528	528	X	X	X	X
HW126_I6	296	550	552	553	553	X	X	445	455
HW127_I7	326	550	541	544	X	X	X	X	X
HW128_I8	302	473	476	478	481	X	424	429	430
HW129_I9	377	541	545	545	546	567	515	542	480

HW130_I10	327	443	442	442	X	X	431	451	437
HW131_I11	305	451	493	484	X	X	431	X	456
HW132_I12	307	454	466	472	X	X	X	X	X
HW133_I13	342	477	495	483	483	X	X	X	X
HW134_I14	271	468	483	483	X	X	X	X	X
HW135_I15	379	537	538	543	543	X	X	X	X
HW141_J6	304	528	529	534	X	X	X	X	X
HW142_J7	295	530	536	545	545	X	X	X	X
HW143_J8	315	454	455	443	X	X	X	X	X
HW144_J9	378	537	544	555	578	578	434	446	446
HW145_J10	314	445	441	442	X	X	X	X	X
HW146_J11	313	436	443	441	X	X	X	X	X
HW147_J12	315	450	441	443	X	X	X	X	X
HW148_J13	290	454	441	443	X	X	X	X	X
HW149_J14	298	481	478	444	X	X	X	X	X
HW150_J15	283	526	527	530	X	X	X	X	X
HW156_K6	303	513	512	520	X	X	X	X	X
HW157_K7	302	517	512	521	X	X	X	X	X
HW158_K8	290	472	496	521	545	536	X	X	X
HW159_K9	375	472	493	521	546	546	X	X	X
HW160_K10	312	466	460	467	X	X	X	X	X
HW161_K11	307	470	441	442	X	X	X	X	X
HW162_K12	310	470	441	443	X	X	X	X	X
HW163_K13	277	456	460	459	X	X	X	X	X
HW164_K14	303	470	460	460	X	X	X	X	X
HW165_K15	342	500	512	521	566	570	X	X	X
HW171_L6	300	570	570	572	578	X	X	X	X
HW172_L7	294	570	563	574	574	571	X	X	X
HW173_L8	318	457	459	460	X	X	X	X	X
HW174_L9	378	582	582	586	589	591	437	450	427
HW175_L10	315	471	485	486	X	X	X	X	X
HW176_L11	308	443	442	443	X	X	439	465	443
HW177_L12	317	445	441	443	X	X	X	X	X
HW178_L13	344	461	459	459	X	X	X	454	447
HW179_L14	320	458	441	443	X	X	X	X	X
HW180_L15	359	562	559	564	564	563	X	X	X
WH017_B2	377	532	535	544	542	X	446	447	441
WH020_B5	250	n/a	440	442	X	X	X	X	X
WH021_B6	366	442	441	442	533	X	X	X	X
WH022_B7	341	445	440	443	X	X	X	X	X
WH023_B8	339	439	441	443	X	X	453	453	453
WH024_B9	348	533	533	542	537	534	X	X	X
WH025_B10	346	461	449	449	X	X	X	X	X
WH027_B12	290	469	448	448	X	X	X	X	X
WH028_B13	340	469	482	486	484	X	X	X	X

WH029_B14	316	444	442	441	X	X	X	X	X
WH030_B15	377	434	442	445	X	X	X	X	X
WH047_D2	378	459	513	538	531	529	455	455	464
WH050_D5	247	X	485	485	X	X	X	X	X
WH051_D6	247	X	448	448	X	X	X	X	X
WH052_D7	307	503	513	512	X	X	515	515	484
WH053_D8	318	441	440	442	X	X	445	445	456
WH054_D9	378	527	528	529	529	530	468	446	468
WH055_D10	315	430	440	443	530	527	460	460	460
WH057_D12	308	473	486	486	X	X	467	467	468
WH058_D13	337	438	438	438	X	X	450	449	467
WH059_D14	315	457	446	446	X	X	446	449	460
WH060_D15	298	481	486	486	486	X	485	X	X
WH062_E2	377	561	561	567	569	568	405	400	446
WH065_E5	285	449	450	450	X	X	X	X	X
WH066_E6	n/a	447	440	440	X	X	X	X	X
WH067_E7	322	539	539	541	545	541	466	458	464
WH068_E8	292	434	440	442	X	X	440	460	448
WH069_E9	302	563	563	564	565	565	449	463	490
WH070_E10	341	429	440	443	X	X	X	X	X
WH072_E12	276	446	441	442	X	X	X	X	X
WH073_E13	330	447	445	444	X	X	X	X	X
WH074_E14	324	446	442	443	X	X	X	X	X
WH075_E15	384	550	550	546	550	X	X	X	X
WH077_F2	376	528	530	533	533	534	426	459	454
WH080_F5	251	483	503	503	506	X	X	X	X
WH081_F6	365	437	441	443	X	X	X	X	X
WH082_F7	288	472	486	485	486	X	X	X	X
WH083_F8	317	433	440	442	X	X	X	X	X
WH084_F9	342	523	525	528	527	527	X	X	X
WH085_F10	338	451	448	448	X	X	X	X	X
WH087_F12	297	475	440	443	X	X	X	X	X
WH088_F13	331	452	444	441	X	X	X	X	X
WH089_F14	306	X	442	443	X	X	X	X	X
WH090_F15	377	513	514	517	516	X	X	X	X
WH107_H2	351	546	546	550	547	546	423	449	442
WH110_H5	311	519	512	511	X	X	X	X	X
WH111_H6	366	444	440	442	539	529	X	X	X
WH112_H7	309	512	512	520	519	X	X	X	X
WH113_H8	313	X	441	442	X	X	X	X	X
WH114_H9	346	543	543	541	543	542	418	453	448
WH115_H10	290	462	442	444	X	X	X	X	X
WH117_H12	308	522	511	521	X	X	X	X	X
WH118_H13	277	443	441	443	X	X	X	X	X
WH119_H14	300	451	441	443	X	X	X	X	X

WH120_H15	382	531	542	533	531	X	X	X	X
WH137_J2	357	547	543	552	544	541	X	X	X
WH140_J5	310	473	468	437	X	X	X	X	X
WH141_J6	276	451	445	446	X	X	X	X	X
WH142_J7	314	500	482	453	X	X	X	X	X
WH143_J8	304	468	455	451	X	X	X	X	X
WH144_J9	358	535	532	543	540	537	X	X	X
WH145_J10	306	457	445	442	X	X	X	X	X
WH147_J12	301	470	454	X	X	X	X	X	X
WH148_J13	340	453	448	443	X	X	X	X	X
WH149_J14	313	454	446	426	X	X	X	X	X
WH150_J15	295	X	456	426	X	X	X	X	X
WH152_K2	346	525	533	542	541	538	433	446	X
WH155_K5	322	478	492	X	X	X	X	X	X
WH156_K6	257	X	X	X	X	X	X	X	X
WH157_K7	303	469	486	446	X	X	X	X	X
WH158_K8	317	430	440	443	X	X	424	433	451
WH159_K9	352	523	523	524	525	525	505	526	479
WH160_K10	344	451	446	444	X	X	X	X	X
WH162_K12	300	502	517	532	530	X	X	X	X
WH163_K13	252	444	442	446	X	X	X	X	X
WH164_K14	324	447	444	437	X	X	X	X	X
WH165_K15	335	504	512	513	513	X	X	X	X
WH167_L2	324	510	513	522	522	522	436	442	436
WH170_L5	252	484	464	X	X	X	X	X	X
WH171_L6	X	505	480	519	534	X	X	X	X
WH172_L7	252	467	478	453	X	X	466	468	435
WH173_L8	251	429	444	447	X	X	422	428	444
WH174_L9	330	521	520	523	523	522	500	X	465
WH175_L10	340	455	459	451	X	X	517	456	441
WH177_L12	327	462	469	446	X	X	X	X	X
WH178_L13	252	445	444	443	X	X	X	X	X
WH179_L14	321	446	445	447	X	X	X	X	X
WH180_L15	259	489	500	501	501	X	X	X	X
WH182_M2	351	419	448	442	X	X	400	400	438
WH185_M5	321	438	445	440	X	X	X	X	X
WH186_M6	377	439	466	449	X	X	410	425	435
WH187_M7	316	556	476	472	X	X	456	456	463
WH188_M8	294	431	445	436	X	X	400	400	450
WH189_M9	338	570	570	570	570	570	450	466	460
WH190_M10	328	426	446	440	X	X	X	429	434
WH192_M12	290	432	452	425	X	X	X	X	X
WH193_M13	336	449	449	458	X	X	423	443	443
WH194_M14	337	435	445	441	X	X	X	X	X
WH195_M15	342	561	513	533	565	X	X	X	X

WH197_N2	359	470	486	479	X	X	X	514	525
WH200_N5	276	470	476	446	X	X	X	X	X
WH201_N6	249	469	452	443	X	X	X	X	X
WH202_N7	308	473	465	448	X	X	X	X	X
WH203_N8	303	502	499	489	505	X	X	X	X
WH204_N9	393	523	513	543	539	527	X	X	X
WH205_N10	349	471	482	482	490	X	X	X	X
WH207_N12	305	465	454	449	X	X	X	X	X
WH208_N13	348	448	448	X	X	X	X	X	X
WH209_N14	337	451	455	448	X	X	X	X	X
WH210_N15	255	482	499	524	538	533	X	X	X

*For crude compounds, X denotes lack of fluorescence emission at the specified wavelength.
*For purified compounds, X denotes the emission maximum of all compounds not purified and screened as well as the compounds that did not demonstrate fluorescence emission at the specified wavelength.
