# Supplementary material for: Structure-Activity Relationship of Nerve-Highlighting Fluorophores
Source: PLoS One. 2013 Sep 9;8(9):e73493. doi: 10.1371/journal.pone.0073493 (PMC3767781; doi:10.1371/journal.pone.0073493)
Supplement: Table S5 — (PDF) [file pone.0073493.s007.pdf]

Table S5 – *Ex Vivo* Assay Results

Fluorophore Name	Crude Fluorophore			Pure Fluorophore
	10 μM	100 μM	1 mM	
HW006_A6	-	-/+	+	-
HW007_A7	-	-/+	-/+	-
HW008_A8	-	-/+	+	-
HW009_A9	+	+	+	+
HW010_A10	+	+ +	+ +	-/+
HW011_A11	+	+	+ +	-/+
HW012_A12	+	+	+	-
HW013_A13	-	-/+	-/+	-/+
HW014_A14	-	-	-/+	-
HW015_A15	-	-	-/+	-
HW021_B6	-	-	-/+	X
HW022_B7	-	-	-/+	X
HW023_B8	-	-/+	-/+	X
HW024_B9	+	+ +	+ +	+ +
HW025_B10	-/+	-/+	+	X
HW026_B11	+	+	+	+
HW027_B12	-	-/+	-/+	X
HW028_B13	-/+	+	+	X
HW029_B14	-	-	-/+	X
HW030_B15	-	-/+	-/+	X
HW036_C6	-	-/+	-/+	X
HW037_C7	-	-	-/+	X
HW038_C8	+	+	+	-
HW039_C9	+ +	+ +	+ +	+ +
HW040_C10	+	+	+	-
HW041_C11	+	+	+	+
HW042_C12	-	-/+	-/+	X
HW043_C13	+	+	+	X
HW044_C14	-	-/+	-/+	X
HW045_C15	-/+	-/+	+	X
HW051_D6	-	-	-/+	X
HW052_D7	-	-/+	-/+	X
HW053_D8	-/+	+	+	X
HW054_D9	+	+ +	+ +	+ +
HW055_D10	-/+	+	-/+	X
HW056_D11	+	+	+	+ +
HW057_D12	-	-/+	-/+	X
HW058_D13	+	+	N/A	X
HW059_D14	-/+	-/+	-/+	X
HW060_D15	-	-/+	-/+	X
HW066_E6	-/+	+	+	X

HW067_E7	-	-/+	+	X
HW068_E8	-/+	+	+	X
HW069_E9	+ +	+ +	+ +	+ +
HW070_E10	-	+	+	X
HW071_E11	+	+	+ +	+
HW072_E12	-	-/+	-/+	X
HW073_E13	+	+	N/A	X
HW074_E14	-	-/+	-/+	X
HW075_E15	-/+	-/+	-/+	-
HW081_F6	-	-/+	-/+	X
HW082_F7	-	-/+	-/+	X
HW083_F8	-/+	+	+	X
HW084_F9	+	+ +	+ +	+
HW085_F10	-	-/+	-/+	X
HW086_F11	-	-/+	-/+	X
HW087_F12	-	-	-/+	X
HW088_F13	-	+	+	X
HW089_F14	-	-	-/+	X
HW090_F15	-	-/+	-/+	X
HW096_G6	-	+	+	X
HW097_G7	-	-	-/+	X
HW098_G8	+	+	+	-/+
HW099_G9	+	+ +	+ +	+ +
HW100_G10	-	-/+	-/+	X
HW101_G11	+	+	+	+
HW102_G12	-	-/+	-/+	X
HW103_G13	-	-	-/+	X
HW104_G14	-	+	-/+	X
HW105_G15	-	-/+	-/+	X
HW111_H6	+	+	+	-
HW112_H7	-	-/+	-/+	X
HW113_H8	+	+	+	-
HW114_H9	+ +	+ +	+ +	+
HW115_H10	+	+	+	-/+
HW116_H11	+	+	+	-
HW117_H12	-	-/+	-/+	X
HW118_H13	+	+	N/A	X
HW119_H14	-	-/+	-/+	X
HW120_H15	-	+	+	X
HW126_I6	+	+	+	-/+
HW127_I7	-	-	-/+	X
HW128_I8	+ +	+ +	N/A	-
HW129_I9	+ +	+ +	+ +	+
HW130_I10	+	+	+	-/+
HW131_I11	+	+ +	+ +	+

HW132_I12	-	-/+	-/+	X
HW133_I13	+	+	N/A	X
HW134_I14	-	-/+	-/+	X
HW135_I15	-	-/+	-/+	X
HW141_J6	-	-/+	-/+	X
HW142_J7	-	-/+	-/+	X
HW143_J8	-	-/+	+	X
HW144_J9	+	+	+	+
HW145_J10	-	-/+	+	X
HW146_J11	-	+	+	X
HW147_J12	-	-/+	-/+	X
HW148_J13	-	-/+	-/+	X
HW149_J14	-	-/+	-/+	X
HW150_J15	-	-/+	-/+	X
HW156_K6	-	-/+	-/+	X
HW157_K7	-	-/+	-/+	X
HW158_K8	-	-/+	-/+	X
HW159_K9	-	-/+	-/+	X
HW160_K10	-	-/+	-/+	X
HW161_K11	-	-/+	-/+	X
HW162_K12	-	-/+	-/+	X
HW163_K13	-	-	-	X
HW164_K14	-	-	-/+	X
HW165_K15	-	-/+	-/+	X
HW171_L6	-	-/+	+	X
HW172_L7	-	-/+	-/+	X
HW173_L8	-	+	-/+	X
HW174_L9	+	+	+	+
HW175_L10	+	+	N/A	X
HW176_L11	+	+	+	+
HW177_L12	-	+	-/+	X
HW178_L13	+	+	+	-/+
HW179_L14	-	+	-/+	X
HW180_L15	-	-/+	-/+	X
WH017_B2	+ +	+ +	+ +	+ +
WH020_B5	-	-	-	X
WH021_B6	+	+	+	N/A
WH022_B7	-	-/+	+	X
WH023_B8	-/+	+	+	X
WH024_B9	+ +	+ +	+ +	+ +
WH025_B10	-/+	+	+	X
WH027_B12	-	-/+	-/+	X
WH028_B13	+ +	+ +	+ +	N/A
WH029_B14	-	-/+	-/+	X
WH030_B15	-	+	-/+	X

WH047_D2	+	+ +	+ +	+
WH050_D5	-	-	-	X
WH051_D6	-	-	-/+	X
WH052_D7	-	-/+	-/+	-/+
WH053_D8	-	-/+	-/+	-/+
WH054_D9	-/+	-/+	+	-/+
WH055_D10	-	-	-/+	+
WH057_D12	-	-	-/+	-/+
WH058_D13	-	-	-/+	-
WH059_D14	-	-	-/+	-
WH060_D15	-	-	-/+	-/+
WH062_E2	+	+ +	+ +	+ +
WH065_E5	-	-/+	N/A	X
WH066_E6	-	-/+	-/+	X
WH067_E7	+	+	+	-/+
WH068_E8	+	+	+	+
WH069_E9	+ +	+ +	+ +	+ +
WH070_E10	-/+	+	+	X
WH072_E12	-	+	-/+	X
WH073_E13	-/+	-/+	-/+	X
WH074_E14	-/+	+	+	X
WH075_E15	-	-/+	-/+	X
WH077_F2	+	+	+	+
WH080_F5	-	-/+	+	X
WH081_F6	-	-	-/+	X
WH082_F7	-/+	-/+	-/+	X
WH083_F8	-	-	-/+	X
WH084_F9	-/+	-/+	-/+	-
WH085_F10	-	-/+	-/+	X
WH087_F12	-	-	-	X
WH088_F13	-	-/+	-/+	X
WH089_F14	-	-	-/+	X
WH090_F15	-	-	-/+	X
WH107_H2	+	+	+	+ +
WH110_H5	-/+	-/+	-/+	X
WH111_H6	-/+	+	+	X
WH112_H7	-/+	+	-/+	X
WH113_H8	-/+	+	-/+	X
WH114_H9	+	+	+	+ +
WH115_H10	-	+	-/+	X
WH117_H12	-	-/+	-/+	X
WH118_H13	-/+	+	+	X
WH119_H14	-	+	-/+	X
WH120_H15	-	+	-/+	X
WH137_J2	-	-/+	+	X

WH140_J5	-	-	-	X
WH141_J6	-	-	-/+	X
WH142_J7	-/+	+	+	X
WH143_J8	-	-/+	-/+	X
WH144_J9	-	-/+	-/+	+
WH145_J10	-	-/+	-/+	X
WH147_J12	-	-/+	-/+	X
WH148_J13	-	-/+	-/+	X
WH149_J14	-	-/+	-/+	X
WH150_J15	-	+	-/+	X
WH152_K2	+ +	+ +	+ +	+
WH155_K5	-	-	-/+	X
WH156_K6	-	-/+	-/+	X
WH157_K7	-/+	+	-/+	X
WH158_K8	+	+	+	-/+
WH159_K9	+	+ +	+ +	+ +
WH160_K10	-	-/+	-/+	X
WH162_K12	-	-/+	-/+	X
WH163_K13	-	+	-/+	X
WH164_K14	-	-/+	-/+	X
WH165_K15	-	-/+	+	X
WH167_L2	+ +	+ +	+ +	+
WH170_L5	-	-	-/+	X
WH171_L6	-	-	-/+	X
WH172_L7	+	+	+	-
WH173_L8	+	+	+	-/+
WH174_L9	+	+	+	-/+
WH175_L10	+	+	+	-
WH177_L12	-/+	+	+	X
WH178_L13	-	-/+	-/+	X
WH179_L14	-/+	+	+	X
WH180_L15	-/+	-/+	-/+	X
WH182_M2	+	+	+	+
WH185_M5	-	-	-/+	X
WH186_M6	+	+	+	-
WH187_M7	+	+	+	-/+
WH188_M8	+	+	+	+
WH189_M9	+ +	+ +	+ +	+ +
WH190_M10	+	+	+	+
WH192_M12	-/+	-/+	-/+	X
WH193_M13	+	+	+	-
WH194_M14	-/+	-/+	-/+	X
WH195_M15	-/+	+	+	X
WH197_N2	+	+	+	+
WH200_N5	-	-	-/+	X

WH201_N6	-	-	-	X
WH202_N7	-	+	+	X
WH203_N8	-	-/+	-/+	X
WH204_N9	-/+	-/+	+	-
WH205_N10	+	+	+	-
WH207_N12	-	-/+	-/+	X
WH208_N13	-	-/+	-/+	X
WH209_N14	-/+	+	+	X
WH210_N15	+	+	+	-

- = Nerve fluorescence equivalent to control autofluorescence
-/+ = Nerve fluorescence lower than BMB, but higher than control
+ = Nerve fluorescence equivalent to BMB with same exposure time
+ + = Nerve fluorescence brighter than BMB with lower exposure time
N/A = Synthetic yield too low to test desired concentration
